# Engineering Two-in-One Nanoparticles for Simultaneous Delivery of Graphene Quantum Dot and Pemetrexed

**DOI:** 10.1021/acsomega.5c07253

**Published:** 2025-10-07

**Authors:** Umut Can Öz, Berrin Küçüktürkmen, I. Jénnifer Gómez, Amr Elsherbeny, Seda Ipek Tekneci, Özgür Eşim, Selin Göksever, Umut Uğur Özköse, Sevgi Gülyüz, Claudia Bazán-Cobelo, Özgür Yılmaz, Aylin Üstündağ, Jiřina Medalová, Asuman Bozkır, Lenka Zajíčková, Engin Er

**Affiliations:** † Department of Pharmaceutical Technology, Faculty of Pharmacy, Ankara University, Yenimahalle, Ankara 06560, Türkiye; ‡ CICA-Centro Interdisciplinar de Química e Bioloxía, Universidade da Coruña, Rúa as Carballeiras, A Coruña 15071, Spain; § Division of Molecular Therapeutics and Formulation, School of Pharmacy, University of Nottingham, Nottingham NG7 2RD, U.K.; ∥ bex Vivo Cancer Pharmacology Centre, Translational Medical Sciences, Biodiscovery Institute, School of Medicine, University of Nottingham, Nottingham NG7 2UH, U.K.; ⊥ Department of Chemical Engineering and Biotechnology, University of Cambridge, Cambridge CB3 0As, U.K.; # Department of Pharmaceutical Toxicology, Faculty of Pharmacy, Ankara University, Yenimahalle, Ankara 06560, Türkiye; ∇ Ankara University Graduate School of Health Sciences, Diskapi, Ankara 06110, Türkiye; ○ Department of Pharmaceutical Technology, Gulhane Faculty of Pharmacy, University of Health Sciences, Etlik, Keçiören, Ankara 06010, Turkey; ◆ Marmara Research Center, TUBITAK, Gebze, Kocaeli 41470, Türkiye; ¶ Department of Chemistry, Faculty of Science and Letters, Piri Reis University, Tuzla, İstanbul 34940, Türkiye; ⟁ Department of Pharmaceutical Chemistry, Faculty of Pharmacy, Istanbul University, Istanbul 34116, Türkiye; △ Department of Experimental Biology, Faculty of Science, Masaryk University, Kamenice 5, Brno 62500, Czech Republic; ▲ Department of Condensed Matter Physics, Faculty of Science, Masaryk University, Brno 61137, Czech Republic; ▽ Central European Institute of Technology − CEITEC, Brno University of Technology, Brno 61200, Czech Republic; ▼ Department of Biotechnology, Biotechnology Institute, Ankara University, Keçiören, Ankara 06135, Türkiye

## Abstract

The simultaneous delivery of therapeutic agents and imaging probes using polymeric nanoparticles (NPs) has gained significant attention for cancer treatment. In this work, we developed a multifunctional nanocarrier system composed of an amphiphilic block copolymer, poly­(2-ethyl-2-oxazoline)-*b*-poly­(ε-caprolactone) (PEtOx-*b*-PCL), and dimethyldidodecylammonium bromide (DDAB), for the codelivery of the chemotherapeutic drug pemetrexed (PMT) and nitrogen- or sulfur-doped graphene quantum dots (N-GQDs or S-GQDs) as fluorescent probes. Critical formulation parameters were optimized using a central composite design (CCD). The optimized NPs exhibited favorable physicochemical properties, including positive surface charge (6–8 mV), hydrodynamic diameters of ∼140 nm, and high encapsulation efficiency for both PMT (46–56%) and GQDs (>98%). *In vitro* assays revealed that PMT-loaded nanoparticles (NPs) significantly enhanced cytotoxicity against MCF-7 cells. At a concentration of 2 ppm after 72 h, N-PMT NPs and S-PMT NPs inhibited cell proliferation by 50.7% and 53.8%, respectively, compared to 37.8% inhibition with free PMT at the same dose. Confocal microscopy confirmed efficient intracellular uptake and strong fluorescence signals, supporting their potential for bioimaging. Collectively, these results demonstrate that this two-in-one nanocarrier system significantly enhances chemotherapeutic efficacy while enabling real-time imaging, establishing a promising platform for drug delivery and noninvasive treatment monitoring in cancer nanomedicine.

## Graphical abstract

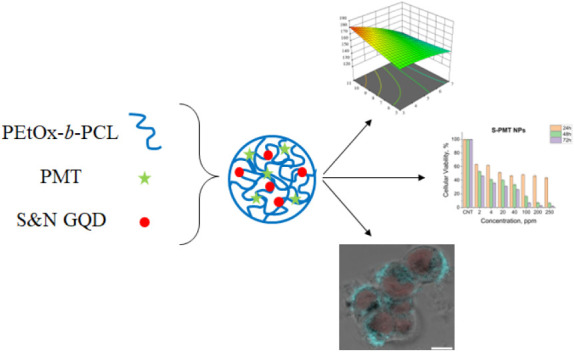

## 1 Introduction

Over the past decade, nanomaterials have become indispensable tools in biomedical research. Nanotechnology, defined as the manipulation of matter at the 1–100 nm scale, has transformed disease diagnosis, therapy, and prevention. Among its most impactful applications is nanoparticle-mediated drug delivery. Nanoparticles can encapsulate therapeutic agents and direct them to pathological sites such as tumors, sites of infection, or inflamed tissues, thereby improving therapeutic efficacy, bioavailability, and controlled release. Several carrier platforms including liposomes, dendrimers, and polymeric nanoparticles have been engineered to enhance the solubility and stability of hydrophobic drugs while minimizing systemic toxicity.^1^ Within polymeric systems, materials such as poly­(lactic acid), polycaprolactone (PCL), poly­(lactic-*co*-glycolic acid), and polyethylene glycol (PEG) have been widely studied for their biocompatibility, tunable release profiles, and amenability to surface modification. In particular, PCL-based nanoparticles, with their slow degradation rate, are attractive candidates for sustained-release drug and nucleic acid delivery.^2–6^

PEGylation is commonly used in nanocarrier design to reduce nonspecific interactions and protein adsorption. However, concerns about PEG-related hypersensitivity and antibody formation have spurred interest in alternative polymers. Poly­(2-oxazoline)­s (POx), and especially poly­(2-ethyl-2-oxazoline) (PEtOx), offer superior hydrophilicity, biocompatibility, and tunable chemistry. POx-based carriers have demonstrated high drug-loading efficiency and self-assembly capacity, making them promising platforms for applications ranging from cancer therapy to bioimaging.^7–9^

Alongside advances in polymeric systems, carbon-based nanomaterials, particularly graphene quantum dots (GQDs), have attracted significant attention. GQDs combine low cytotoxicity, excellent biocompatibility, water solubility, and unique photoluminescent properties with cost-effective synthesis routes.^10–13^ These characteristics make them suitable for diverse applications,^14,15^ including bioimaging,^16–18^ optoelectronics,^19^ sensing, and photocatalysis.^18,20–22^ Structural modifications, such as heteroatom doping, further enhance their optical and electronic properties.^23–25^ Nitrogen- and sulfur-doped GQDs (N-GQDs and S-GQDs) are especially promising for biomedical applications due to their high surface reactivity, efficient drug-loading capacity, and photoluminescence, enabling simultaneous therapeutic and imaging functions.^26–29^

Pemetrexed (PMT) is a multitargeted antifolate that inhibits key enzymes in folate metabolism and DNA synthesis, ultimately triggering tumor cell apoptosis.^30,31^ Although its primary clinical use has been in nonsmall cell lung cancer and mesothelioma,^32^ emerging evidence supports its activity in breast cancer, particularly in subtypes characterized by high thymidylate synthase expression and in tumors resistant to anthracyclines or taxanes.^33,34^ These features make PMT attractive for repositioning in breast cancer, where treatment options can be limited in resistant disease. Moreover, its manageable toxicity profile enhances its suitability for incorporation into nanoparticle-based delivery systems, which may further improve therapeutic efficacy while minimizing systemic adverse effects.

Despite notable advances, nanoparticle-based theranostic systems continue to face several challenges. Many reported formulations demonstrate either limited drug encapsulation efficiency, insufficient control over critical quality attributes, or a lack of integrated diagnostic functionalities. In particular, PMT-loaded nanocarriers have largely been developed without concurrent imaging capability, whereas GQD-based systems are rarely optimized through statistically robust design approaches. These gaps hinder the translation of such platforms into clinically relevant applications. To address these limitations, we developed a multifunctional nanocarrier that enables simultaneous PMT delivery and bioimaging through N-GQDs or S-GQDs, with formulation parameters systematically optimized using a Quality by Design (QbD) framework.

This study introduces a novel theranostic nanoparticle system that, for the first time, integrates N-GQDs and S-GQDs with a PEtOx-*b*-PCL copolymer matrix for the codelivery of PMT. Unlike previous formulations that separately address drug delivery and imaging, our approach enables simultaneous chemotherapy and bioimaging within a single platform. Furthermore, we employed a QbD-driven Central Composite Design (CCD) to systematically optimize formulation parameters, resulting in potent cytotoxicity against MCF-7 cells at low PMT doses. These features distinguish our system from earlier PMT-loaded nanoparticles, which often lacked either integrated imaging capabilities or rigorous statistical optimization.

## 2 Materials and Methodology

### 2.1 Materials

PMT was purchased from TCI. Dialysis membrane tubes with a cutoff of 0.5–1 kDa were provided by Spectrum Laboratories. 2-Ethyl-2-oxazoline (EtOx, 98%), methyl p-toluenesulfonate (MeTos), and tin­(II) 2-ethylhexanoate (Sn­(Oct)_2_) (92.5–100%) were supplied by Sigma-Aldrich. ε-Caprolactone (ε-CL, 97%) and propargyl alcohol were provided by Acros Organics. All solvents were purchased from Sigma-Aldrich unless otherwise stated. All chemicals were used as received unless otherwise stated.

### 2.2 Polymer Synthesis

#### 2.2.1 Synthesis of Azide-Functionalized Poly­(2-ethyl-2-oxazoline) (PEtOx-N_3_)

Living cationic ring-opening polymerization (LCROP) of 2-ethyl-2-oxazoline (EtOx, 99.06 mmol, 10 mL) was carried out by initiated with methyl *p*-toluenesulfonate (MeTos, 2.89 mmol, 436 μL). Thirty mL of dry acetonitrile was used to solubilize all components, and the reaction was performed under inert conditions at 130 °C for 16 h. Upon completion, quenching of the polymerization with sodium azide (NaN_3_, 11.56 mmol, 752 mg) was accomplished at 65 °C for 24 h. The solvent was subsequently removed under vacuum, and the crude polymer was redissolved in dichloromethane (DCM). Residual sodium azide was eliminated by filtration. The polymer was then precipitated by the dropwise addition of the DCM solution into ice-cold diethyl ether. The resulting precipitate was isolated by decanting the supernatant and consequently dried under reduced pressure overnight, affording 8.5 g of azide-functionalized PEtOx. Yield ∼71%; *M*_n,NMR_ = 4550 g·mol^–1^; *M*_n,GPC_= 4100 g·mol^–1^; dispersity index (Đ = *M*_w_/*M*_n_) = 1.36. IR (υ_max_): 2106 (−N_3_) and 1626 (−NHCO−) cm^–1^. ^1^H NMR (600 MHz, CDCl_3_, δ, ppm, TMS): 3.5–3.3 (m, 4H, −NC ***H***_***2***_ *C* **H**_***2***_– of the polymer backbone), 3.0–2.9 (m, 3H, C ***H***_***3***_ from MeTos), 2.4–2.2 (m, 2H, C ***H***_***2***_ from side group), 1.1–0.9 (m, 3H, C ***H***_***3***_ of the polymer backbone).

#### 2.2.2 Synthesis of Alkyne-Functionalized Poly­(caprolactone) (PCL-yne)

Alkyne-functionalized poly­(ε-caprolactone) (PCL-yne) was acquired through ring-opening polymerization (ROP) of ε-caprolactone (5 mL, 0.045 mol), initiated by propargyl alcohol (19 μL, 0.322 mmol) and catalyzed by tin­(II) 2-ethylhexanoate (Sn­(Oct)_2_, 434 μL, 0.039 mmol) in 5 mL of dry toluene under a nitrogen atmosphere at 120 °C for 4.5 h. Upon completion of the polymerization, the reaction mixture was allowed to cool to room temperature to terminate the reaction. The crude polymer was purified by dissolution in dichloromethane (DCM) followed by dropwise precipitation into cold *n*-hexane. The resulting precipitate was collected by filtration and the final product was dried under reduced pressure. Yield = 3.9 g, 89%; *M*_n,NMR_ = 11072 g·mol^–1^, *M*_n,GPC_ = 13500 g·mol^–1^; dispersity index (Đ = *M*_w_/*M*_n_ = 1.32. IR (υ_max_): 2125 (−C≡CH), 1724 (ester carbonyl (C = O)) cm^–1^. ^1^H NMR (600 MHz, CDCl_3_, δ, ppm): 4.66 (s, 2H, C ***H***_***2***_–C≡CH), 4.00 (m, 2H, -C ***H***_***2***_OC­(O)−), 3.65 (t, 2H, C ***H***_***2***_OH), 2.50 (s, 1H, CH_2_–C≡C ***H***), 2.35–2.27 (m, 2H, -OC­(O)­C ***H***_***2***_), 1.67–1.57 (m, 4H, -OC­(O)­CH_2_C ***H***_***2***_-, -C ***H***_***2***_CH_2_OC­(O)−), 1.40–1.38 (m, 2H, −CH_2_C ***H***_***2***_CH_2_−).

#### 2.2.3 Synthesis of Poly­(2-ethyl-2-oxazoline)-*b*-poly­(ε-caprolactone)­Poly­(ε-Caprolactone) (PEtOx-*B*-PCL)

The amphiphilic block copolymer PEtOx-*b*-PCL was obtained through a copper-catalyzed azide–alkyne cycloaddition (CuAAC) reaction between azide-ended poly­(2-ethyl-2-oxazoline) (PEtOx-N_3_, 0.45 g, 0.12 mmol) and alkyne-ended poly­(ε-caprolactone) (PCL-yne, 1.5 g, 0.11 mmol) by catalyzed with copper­(II) sulfate pentahydrate (CuSO_4_·5H_2_O, 0.018 g, 0.10 mmol) and sodium ascorbate (NaAsc, 0.095 g, 0.6 mmol) in 15 mL of dry DCM under an inert atmosphere at the room temperature for 24 h. Upon completion of the reaction, the crude product was purified by dialysis against distilled water containing ethylenediaminetetraacetic acid (EDTA) using cellulose dialysis tubing (molecular weight cutoff ∼ 6500 Da) for 3 days to remove residual copper ions and low molecular weight impurities. The final product was attained by lyophilization of the dialyzed solution (yield = 1.2 g, 63%). *M*_n,GPC_ = 17200 g·mol^–1^; dispersity index (Đ) = 1.41. IR (υ_max_): 2945, 2865 (C–H stretching vibrations); 1724 (ester carbonyl stretching of the PCL), 1626 (amide carbonyl stretching of PEtOx); no residual N_3_ or alkyne functional group vibrations were observed after the block copolymer reaction. ^1^H NMR (600 MHz, CDCl_3_, δ, ppm): 8.1 (s, 1H, C_2_ ***H*** N_3_), 5.2 (s, 2H, – C ***H***_***2***_C_2_HN_3_), 4.00 (m, 2H, -C ***H***_***2***_OC­(O)−), 3.5–3.3 (m, 4H, – NC ***H***_***2***_ *C* **H**_***2***_– of PEtOx backbone), 2.4–2.2 (m, 2H, C ***H***_***2***_ from PEtOx side group, -OC­(O)­C ***H***_***2***_), 1.67–1.57 (m, 4H, -OC­(O)­CH_2_C ***H***_***2***_-, -C ***H***_***2***_CH_2_OC­(O)−), 1.40–1.38 (m, 2H, −CH_2_C ***H***_***2***_CH_2_−), 1.1–0.9 (m, 3H, C ***H***_***3***_ of PEtOx backbone).

### 2.3 Synthesis of N-GQDs and S-GQDs

N-GQDs and S-GQDs were synthesized by microwave-assisted of a previously established hydrothermal approach in a Discover SP microwave (CEM, USA).^35^ Briefly, 25 mg of glucose dissolved in a 1 mL of ultrapure water was mixed with an ethylenediamine (21.40 μL) solution under stirring. Afterward, the solution was subjected to microwave irradiation under dynamic control mode in a 10 mL Pyrex vessel. Under these conditions, the internal temperature reached up to 200 °C and the pressure up to 200 PSI, with an irradiation time of 150 s (nominal maximum power 200 W) for the growth of N-GQDs. It should be noted that, due to the dynamic control, both temperature and pressure fluctuated during the process and the microwave power was automatically adjusted rather than applied continuously at the nominal value. Subsequently, N-GQDs solution was filtered through a microporous filter (0.1 μm) to eliminate any excess of unreacted compounds. The resulting N-GQDs were then dialyzed using a cutoff membrane filter (0.5–1 kDa) against ultrapure water for 2 days.

S-GQDs were synthesized with a slight modification to the above procedure.^36^ Glucose (25 mg) was dissolved in 1.25 mL of Milli-Q water and followed by the addition of cysteamine hydrochloride (63.62 mg). The solution was then irradiated under dynamic control mode at up to 200 °C and 200 PSI for 180 s (nominal maximum power 250 W). As in the N-GQDs synthesis, the applied microwave power was automatically regulated and not continuously delivered at the nominal set point. The resulting S-GQDs were then subjected to dialysis against an ultrapure water using a cutoff membrane filter (0.5–1 kDa) for 2 days.

### 2.4 Characterization of Polymers and *N*-/S-Doped Graphene Quantum Dots

The detailed characterization methods for polymers and *N*-/S-doped GQDs were presented in Supporting Information file.

### 2.5 Quality by Design Approach for Optimization of PMT-Loaded PEtOx-*b*-PCL Nanoparticles

The experimental design for optimizing PMT loaded PEtOx-*b*-PCL nanoparticles (PMT NPs) was established via Design-Expert software (Version 12.0, Stat-Ease Inc.). Three factors were identified as the critical material attributes (CMAs): the mass ratio of PEtOx-*b*-PCL/DDAB (*X*_1_), the volume ratio of the primary W_1_/O emulsion (*X*_2_), and the volume of the hardening solution (*X*_3_). These factors were chosen to investigate their effects on selected Critical Quality Attributes (CQAs), including the hydrodynamic diameter (*Y*_1_), polydispersity index (PDI, *Y*_2_), and encapsulation efficiency of PMT (*Y*_3_) (Table 1). To ensure a comprehensive exploration of the design space while facilitating practical NP processing, the levels of independent variables were carefully selected. Using Response Surface Methodology in conjunction with the Central Composite Design (CCD) approach, the formulation parameters were explored at five levels (-α, −1, 0, + 1, + α), with an α value of 1.68 chosen for design orthogonality and rotatability (Table 1).

**Table 1:** Factors (Independent) and Response (Dependent) Variables during the Optimization of PMT NPs

	level				
independent variable	–1.68	–1	0	+1	+1.68
mass ratio of PEtOx-*b*-PCL/DDAB (*X*_1_)	2.27	5	9	13	15.73
volume ratio of *W*_1_/*O* phase (*X*_2_)	1.64	3	5	7	8.36
volume of PVA solution (*X*_3_)	2.95	5	8	11	13.04

dependent variable	goal
hydrodynamic diameter, nm (*Y*_1_)	minimize
PDI (*Y*_2_)	minimize
encapsulation efficiency, % (*Y*_3_)	maximize

A total of 20 experimental runs were generated using statistical design software, comprising 6 axial points, 8 factorial points, and 6 center point replications (Table 2). These runs were performed randomly to reduce bias and improve the reliability of the model. All measurements were performed in triplicate (*n* = 3). The response data were fitted to linear, two-factor interaction (2FI), and quadratic models. The polynomial equations derived from these models were validated statistically through analysis of variance (ANOVA). To evaluate the significance of the models, we analyzed statistical parameters, including p-values, adjusted R^2^ (*R* ^2^ *-adj*), and predicted R^2^ (*R* ^2^ *-pred*). Additionally, 3D response surface plots were generated to visually examine the results and explore the interactions between different factors for each response. Target responses were established to achieve the smallest hydrodynamic diameter, the lowest polydispersity index (PDI), and the highest encapsulation efficiency (EE%) in order to optimize the nanoparticle formulation. The optimal independent variables identified through the analysis were then applied to prepare the ideal PMT NPs formulation.

**Table 2:** Experimental Design Points and Measured Values of Responses for Optimizing of PMT NPs

	experimental factors			responses		
formulation	*X*_1_ (w/w)	*X*_2_ (v/v)	*X*_3_ (mL)	*Y*_1_ (nm)	*Y*_2_	*Y*_3_ (%)
1	5	3	5	159.50	0.19	38.25
2	13	3	5	160.80	0.18	49.71
3	5	7	5	139.50	0.26	27.62
4	13	7	5	154.30	0.18	37.55
5	5	3	11	161.50	0.25	15.72
6	13	3	11	183.20	0.27	20.58
7	5	7	11	133.90	0.25	29.67
8	13	7	11	127.80	0.14	33.57
9	2.27	5	8	161.50	0.32	22.72
10	15.73	5	8	161.60	0.22	44.21
11	9	1.64	8	175.70	0.28	40.13
12	9	8.36	8	131.60	0.15	30.61
13	9	5	2.95	157.50	0.18	56.93
14	9	5	13.04	172.70	0.26	23.42
15	9	5	8	158.10	0.18	28.70
16	9	5	8	158.50	0.17	28.40
17	9	5	8	157.80	0.18	29.10
18	9	5	8	159.10	0.18	28.20
19	9	5	8	158.50	0.18	27.90
20	9	5	8	158.20	0.17	28.80

### 2.6 Preparation of PMT- and GQD-Loaded NPs

PMT NPs were fabricated employing a modified double emulsion (water-in-oil-in-water) solvent evaporation technique.^37^ Initially, 1 mg of PMT was dispersed in 70 μL of pH 7.4 Tris buffer solution, creating the W_1_ phase. This solution was combined with 0.5 mL of CHCl_3_ as the O phase, containing varying mass ratios of PEtOx-*b*-PCL copolymer and DDAB cationic lipid on an ice bath using a probe-sonicator (Bandelin Sonopuls HD2070) for 30 s at a power of 35W, resulting in the formation of the W_1_/O emulsion. Subsequently, this emulsion was added onto a 1% poly­(vinyl alcohol) (PVA) solution (W_2_ phase) with varying volumes of O/W_2_ and sonicated (35W) on an ice bath for another 30 s to obtain the W_1_/O/W_2_ emulsion. The final emulsion was injected rapidly into 18 mL of 0.5% PVA hardening solution under stirring, taking care to avoid bubble formation. Following 2.5 h after injection, the NPs were centrifuged at 30,000 rpm for 1 h and collected as a pellet. The pellet was resuspended in the 18 mL of deionized water and centrifuged again under the same conditions. This washing step was repeated three times to remove unencapsulated materials and residual solvent.

To prepare PMT NPs encapsulating GQDs, 20 μL (45 mg/mL) of either N-GQDs or S-GQDs was introduced into the W_1_ phase of the previously described nanoparticle preparation process. The procedure was then continued as outlined, yielding N-PMT NPs or S-PMT NPs, depending on the type of GQDs used.

### 2.7 Characterization of PMT- and GQD-Loaded PMT NPs

#### 2.7.1 Hydrodynamic Diameter, Polydispersity Index (PDI), and Zeta Potential

Dynamic light scattering (DLS) measurements were performed using a Zetasizer Nano instrument (Malvern Instruments, Malvern UK) to assess the hydrodynamic diameter and PDI. All measurements were conducted at ambient temperature, utilizing a scattering angle of 173° and a laser source operating at a wavelength of 633 nm.^38,39^ The zeta potential of the PMT NPs was also analyzed using the Zetasizer Nano system. These zeta potential assessments reveal information about the surface charge characteristics of the NPs, based on the techniques of laser Doppler electrophoresis and phase analysis light scattering. All measurements were conducted with six replicates to ensure statistical reliability, and mean values along with standard deviations (±SD) are reported to indicate the variability within the data.

Additionally, to assess the physical stability of the NPs under physiologically relevant conditions, the formulations were dispersed in Dulbecco’s Modified Eagle Medium (DMEM supplemented with 10% fetal bovine serum) and their hydrodynamic size distribution was monitored by DLS over 24 h at 37 °C.

#### 2.7.2 Transmission Electron Microscopy

A transmission electron microscope (TEM, FEI Tecnai G2 Spirit) was employed to visualize the morphology of the PMT NPs. Briefly, 10 μL of nanoparticle dispersion was dropped onto 200 mesh carbon coated copper grid, and excess amount was blotted using a filter paper after 5 min of incubation. All samples were dried at room temperature before imaging at 120 kV.

#### 2.7.3 Encapsulation Efficiency Determination of PMT

The determination of the encapsulated PMT amount was conducted using an HPLC method. Initially, the prepared PMT NPs were centrifuged at 30,000 rpm, and the supernatant was carefully collected. Subsequently, the supernatant from the nanoparticle dispersion was filtered through a 0.45 μm syringe filter. Quantification of the PMT in the sample was carried out using an HPLC instrument equipped with a Diode Array Detector (DAD) set at a wavelength of 245 nm and a C18 column (Kinetex EVO 5 μm, 250 × 4.6 mm). The mobile phase comprised an acetonitrile (ACN): buffer mixture (30:70), and the injection volume was set at 20 μL, with the buffer consisting of 15 mM pH 3.0 phosphate buffer. The analysis was performed with a flow rate of 0.8 mL/min over a duration of 4 min, conducted in triplicate.

The method was validated according to International Council for Harmonisation (ICH) guidelines. Linearity was confirmed in the concentration range of 0.9–250 μg/mL, with a correlation coefficient (R^2^ = 0.9994). The limit of detection (LOD) and limit of quantification (LOQ) were determined to be 8.5 μg/mL and 25.9 μg/mL, respectively.

The encapsulation efficiency of PMT (EE_PMT_) was calculated using the formula

$${\mathrm{EE}}_{\mathrm{PMT}}=\frac{{\mathrm{PMT}}_{\mathrm{total}}-{\mathrm{PMT}}_{\mathrm{supernatant}}}{{\mathrm{PMT}}_{\mathrm{total}}}\times 100\%$$

where PMT_total_ is the total amount of PMT and PMT_supernatant_ is the amount of PMT in the supernatant.

#### 2.7.4 Encapsulation Efficiency of N-GQD and S-GQD in PMT NPs

The encapsulation efficiencies of the N-GQD and the S-GQD were ascertained through the centrifugation of the NPs at a velocity of 30,000 rpm for 1 h. The quantities of GQD that were not encapsulated were quantitatively assessed using Agilent Cary Eclipse Fluorescence Spectrophotometer. This analysis was conducted at excitation wavelengths of 380 nm for N-GQD and 350 nm for S-GQD, with corresponding emission scanning ranges spanning from 390 to 750 nm for N-GQD and 360 to 750 nm for S-GQD. The encapsulation efficiencies of the N-GQD and the S-GQD were calculated using the formula

$${\mathrm{EE}}_{\mathrm{GQD}}=\frac{{\mathrm{GQD}}_{\mathrm{total}}-{\mathrm{GQD}}_{\mathrm{supernatant}}}{{\mathrm{GQD}}_{\mathrm{total}}}\times 100\%$$

where GQD_total_ is the total amount of N- or S-GQD and GQD_supernatant_ is the amount of N- or S-GQD in the supernatant.

#### 2.7.5 Release Profile of PMT NPs

The release of PMT from the PMT NPs formulation was evaluated using the dialysis method.^40^ Briefly, PMT nanoparticles (PMT concentration 1 mg/mL) and free PMT (1 mg/mL) were loaded into dialysis bags (MWCO: 12–14 kDa, Spectra/Por) and immersed in 25 mL of two different release media: phosphate buffered saline (PBS, pH 7.4) and MES-buffered saline (pH 5.5). The bags were incubated at 37 °C in a shaking water bath (50 rpm) for 96 h. At predetermined time points, 1 mL aliquots of the release medium were withdrawn and immediately replaced with an equal volume of fresh buffer. The amount of DOX released was quantified by HPLC. Cumulative release (%) was then plotted against time (hours) to construct the release profile of DOX from PMT NPs.

### 2.8 Cell Culture and Cell Metabolic Studies

The human breast adenocarcinoma cell line MCF-7 (ATCC HTB-22, Manassas, VA, USA) was cultured in high-glucose Dulbecco’s Modified Eagle Medium (DMEM; Gibco, Cat. No. 11965092) supplemented with 10% fetal bovine serum (FBS; Gibco, Cat. No. 26140079) and 1% penicillin–streptomycin (Gibco, Cat. No. 15140122). Cells were maintained at 37 °C in a humidified atmosphere with 5% CO_2_. Upon reaching approximately 80% confluency, cells were subcultured according to the supplier’s protocol to maintain optimal growth conditions.

For the *in vitro* cytotoxicity assessment, MCF-7 cells were seeded onto ThermoScientific Nunc MicroWell 96-Well Optical-Bottom black plates at a density of 7.0 × 10^3^ cells per well and cultured in DMEM for 24 h. Following this, the culture media was replaced, and the cells were exposed to various concentrations of blank NPs, N-PMT NPs, and S-PMT NPs, as well as equivalent concentrations of free PMT, N-GQDs, S-GQDs, or a negative control (cell culture grade water) for 24, 48, and 72 h. Free PMT served as the positive control. Subsequently, the treatments were removed, and the cells were subjected to the MTT assay (Sigma-Aldrich, Cat. No. M5655; incubated for 4 h at 37 °C) to evaluate their metabolic activity. Metabolic activity was assessed by measuring absorbance using a ThermoScientific Multiskan Go microplate reader at 540 nm.

### 2.9 Confocal Microscopy Analyses

Localization of two-in-one nanoparticles was studied by confocal fluorescence by Zeiss LSM 880 laser scanning microscope with an Airyscan-Fast module (LSM880_Airy-A2, Zeiss, Germany). The NPs fluorescence was taken in λ_ex_ = 405 nm and λ_em_ = 415–600 nm. The nuclear stain DRAQ5 (Cell Signaling Technology, Cat. No. 4084) imaging was carried out using λ_ex_ = 633 nm and λ_em_ = 650–735 nm. Briefly, 2 × 10^4^ MCF7 cells (ATCC, Manassas, VA) were seeded in 200 μL to an μ-Slide 8 Well (IBIDI GmbH, Gräfelfing, Bayern, Germany) and cultivated for 24 h. Then 600 μg/mL of QD was added and in 5 h were cells stained by the 5 μM DNA intercalating stain DRAQ5 (Cell Signaling, Danvers, MA) for at least 5 min.

### 2.10 Statistical Analyses

Data are presented as mean ± standard deviation. Statistical analysis was performed using a one-way ANOVA followed by Tukey’s post hoc multiple comparisons test. Results are reported as not significant (NS) or significant at the following thresholds: *p* < 0.05 (*), *p* < 0.01 (**), *p* < 0.001 (***), and *p* < 0.0001 (****).

## 3 Results and Discussion

### 3.1 Polymer Synthesis and Characterization

In this study, PEtOx-*b*-PCL block copolymer was synthesized to enable the formation of polymeric nanoparticles capable of coloading and delivery of PMT and either N-GQDs or S-GQDs. PEtOx, selected as the hydrophilic head, possesses high water solubility, low toxicity, stability, and excellent biocompatibility, making it well-suited for drug delivery systems. Meanwhile, PCL was selected as the hydrophobic tail owing to its favorable biocompatibility and biodegradability, properties that are essential for its function as the hydrophobic core in micellar structures designed for the encapsulation of hydrophobic therapeutic agents such as PMT. This combination of PEtOx and PCL in block copolymers or nanoparticle systems provides versatility as drug delivery platforms, leveraging the favorable properties of both polymers.

The copper-catalyzed azide–alkyne cycloaddition (CuAAC) reaction between azide-functionalized poly­(2-ethyl-2-oxazoline) (PEtOx-N_3_) and alkyne-functionalized poly­(ε-caprolactone) (PCL-yne) was succeeded to yield the amphiphilic block copolymer PEtOx-*b*-PCL. Both precursor polymers were obtained in high yields with well-controlled molecular weights, as confirmed by the results of their respective polymerization reactions.

The PEtOx-N_3_ precursor was synthesized by living cationic ring-opening polymerization (LCROP) of 2-ethyl-2-oxazoline, and PCL-yne by tin­(II) 2-ethylhexanoate catalyzed ring-opening polymerization of ε-caprolactone. ^1^H NMR spectra of these precursors (Figure 1a,b) display the expected backbone resonances and well resolved end-group signals. Gel permeation chromatography and Fourier-transform infrared spectroscopy data, as previously reported earlier,^37^ confirm narrow dispersities (Đ ≈ 1.36 for PEtOx-N_3_, Đ ≈ 1.32 for PCL-yne and Đ ≈ 1.41 for PEtOx-*b*-PCL) and demonstrate the presence of azide (2106 cm^–1^), alkyne (2125 cm^–1^) and ester carbonyl (1724 cm^– 1^) functionalities, thereby attesting to controlled molecular weights and preserved functional group integrity. ^1^H NMR analysis of the coupling product (Figure 1c) reveals complete disappearance of the azide (−N_3_) and terminal alkyne resonances and the emergence of distinct triazole proton signals at δ 8.10 and 5.20 ppm, alongside the characteristic methylene and methyl resonances of the PEtOx and PCL segments, confirming quantitative cycloaddition. The combination of spectroscopic and chromatographic techniques unequivocally confirms the successful formation of well-defined PEtOx-*b*-PCL block copolymers, exhibiting controlled block lengths and a narrow molecular weight distribution.

**Figure 1:**
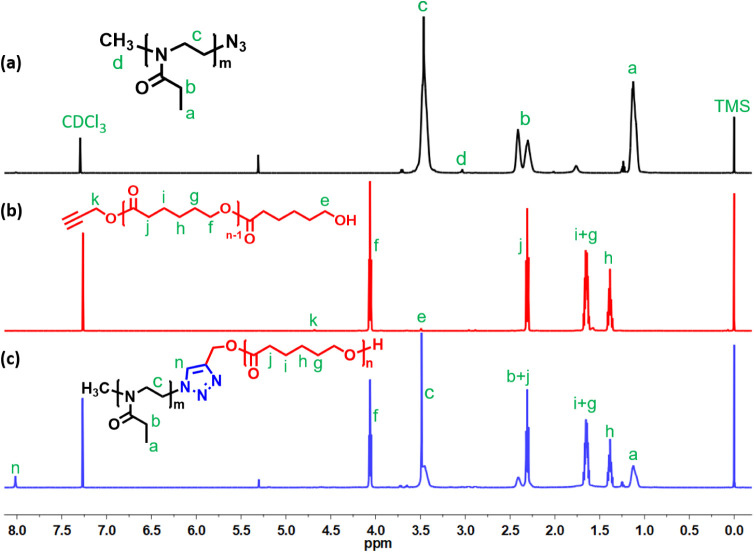
^1^H NMR spectra of the PEtOx homopolymer (a), PLC homopolymer (b), and PEtOx-*b*-PCL copolymer (c).

### 3.2 Synthesis and Characterization of N-GQDs and S-GQDs

N-GQDs were synthesized via our previously reported low-cost, bottom-up microwave-assisted method under controlled conditions.^35^ To produce sulfur-doped GQDs (S-GQDs), this protocol was slightly adapted^36^ by using glucose and cysteamine as the carbon and sulfur precursors, respectively. Both *N*- and S-GQDs exhibited quasi-spherical morphology with sizes below 10 nm as observed in Figures S1 and S8, with an average size of 4.15 ± 1.73 nm for N-GQDs and 4.44 ± 2.26 nm for S-GQDs, respectively. Furthermore, the as-prepared dots presented a variety of oxygen-, nitrogen-, and sulfur-based surface moieties (Tables S1–S2, Figures S2–S5 and S9–S13). Furthermore, the as-prepared GQDs exhibited excitation wavelength-dependent multicolor emissions with a maximum of λ_ex_ 380 nm for N-GQDs and λ_ex_ 340 nm for S-GQDs (further details in Supporting Information).

### 3.3 Preparation and Optimization of PMT NPs Using a QbD Approach

PMT NPs were successfully synthesized using a water-in-oil-in-water (W_1_/O/W_2_) double-emulsion technique, a widely recognized method for producing stable nanoparticles with uniform size distribution.^41^ In this process, DDAB was used as a cationic lipid surfactant in stabilizing the primary water-in-oil (W_1_/O) emulsion, while PVA functioned as a nonionic surfactant to stabilize the secondary water-in-oil–water (W_1_/O/W_2_) nanoparticle emulsion.^42,43^

In this study, the Quality Target Product Profile (QTPP) for PMT/PEtOx-*b*-PCL NPs was defined with specific quality attributes focusing on achieving the smallest hydrodynamic diameter and PDI while maximizing % encapsulation efficiency (EE). To optimize the PMT NPs, Design of Experiments (DoE) and Quality by Design (QbD) principles were employed. A Central Composite Design (CCD) method in combination with Response Surface Methodology was utilized to generate 20 formulations of PMT NPs (Table 2). The optimization process centered on critical material attributes (CMAs), such as the mass ratio of PEtOx-*b*-PCL to DDAB (*X*_1_), the volume ratio of the W_1_/O phase (*X*_2_), and the volume of the PVA solution (*X*_3_), which had previously been identified as influential factors in determining the QTPP of the final NP formulation.^44,45^

The study focused on evaluating the impact of these CMAs on key parameters including hydrodynamic diameter (*Y*_1_), PDI (*Y*_2_), and PMT EE (*Y*_3_). Model fitting was carried out using Design Expert software (Version 12), evaluating linear, two-factor interaction (2FI), quadratic, and cubic models. Statistical evaluations indicated that the second-order (quadratic) polynomial model provided the best fit for all response variables (Table 3). The high level of agreement between predicted and observed results was evidenced by minimal differences (less than 0.2) between *R* ^2^-pred and *R* ^2^-adj values across all responses (Table 3).^46,47^ Moreover, all responses exhibited adequate precision values above 4, supporting the appropriateness of the selected models for navigating the design space (Table 3).^46,47^

**Table 3:** ANOVA Findings Corresponding to the Second-Order Polynomial Model for All Response Variables

	ANOVA results				
dependent variable	*F*	*P*	*R* ^2^	adj *R* ^2^	Adeq precision
hydrodynamic diameter, nm (*Y*_1_)	8.69	0.0072	0.9496	0.8403	12.6061
PDI (*Y*_2_)	10.93	0.0039	0.9595	0.8717	11.3491
encapsulation efficiency, % (*Y*_3_)	9.37	0.0059	0.9531	0.8513	11.2763

A mathematical model in the form of a second-order polynomial was constructed based on the analysis:

$$Y=\beta_{ο}+\beta_{1}X_{1}+\beta_{2}X_{2}+\beta_{3}X_{3}+\beta_{11}X_{1}^{2}+\beta_{22}X_{2}^{2}+\beta_{33}X_{3}^{2}+\beta_{12}X_{1}X_{2}+\beta_{13}X_{1}X_{3}+\beta_{23}X_{2}X_{3}$$

where Y is the response, β_0_ represents the intercept coefficient, β_1_, β_2_, β_3_, ..., β_23_ correspond to the regression coefficients linked to the factors X_1_, X_2_, X_3_, and so forth, each at their designated levels. The terms X_1_X_2_, X_1_X_3_, and X_2_X_3_ depict the interaction effects between different factors, while X_1_^2^, X_2_^2^, and X_3_^2^ indicate the quadratic terms. Utilizing coded factors, this equation enables the prediction of the response based on the specified levels of each factor. Moreover, it facilitates the evaluation of the factors’ relative impact by comparing their regression coefficients. A positive coefficient value signifies a synergistic impact, whereas a negative one implies an antagonistic effect between the independent and dependent variables.^46,47^ To determine the model’s significance, we assessed the *P*-value, considering a value below 0.05 as indicative of the statistical significance of the derived equation.

#### 3.3.1 Hydrodynamic Diameter

The hydrodynamic diameter of nanoparticles significantly influences their interaction with biological systems. Research has shown that hydrodynamic diameter impacts cell uptake, encapsulation efficiency of therapeutic agents, and pharmacokinetic profile of the drug delivery system. NPs of smaller sizes play a crucial role in enhancing drug delivery efficiency and therapeutic outcomes. Research indicates that smaller nanoparticles are less likely to be recognized and eliminated by the mononuclear phagocyte system (MPS), allowing for prolonged circulation times and increased chances of reaching target cells.^48^ Moreover, smaller nanoparticles exhibit greater tumor penetration depths, which is essential for effective drug delivery to tumor sites.^49^ Studies have shown that nanoparticles smaller than 300 nm can effectively enter target cells to exert their pharmacological functions, highlighting the importance of nanoparticle size in achieving desired therapeutic effects.^50^ Additionally, smaller nanoparticles have been associated with enhanced bioavailability, increased aqueous dispersibility, and improved tissue distribution, all of which contribute to their effectiveness in drug delivery systems.^51^ As such, it was imperative to include the hydrodynamic diameter as a QTTP parameter in our optimization.

The mean hydrodynamic diameter, expressed as the z-average diameter, varied between 127.80 and 183.20 nm across the 20 formulations, as shown in Table 2. The differences in the responses across the formulations indicates that the studied CMAs contributed to changes on the analyzed response variables. The positive coefficients of the mass ratio of PEtOx-*b*-PCL/DDAB (*X*_1_) and volume of PVA solution (*X*_3_) in the polynomial equation suggested that increasing the mass ratio of PEtOx-*b*-PCL/DDAB or the volume of the PVA solution increased the hydrodynamic diameter of the NPs, however these effects were not statistically significant (*P*-value = 0.9903 and 0.1029, respectively). On the other hand, the negative coefficient of the volume ratio of W_1_/O phase (*X*_2_) indicated that the hydrodynamic diameter decreased with an increase in the volume ratio, a relationship which proved statistically significant (*P*-value = 0.0014). This finding is consistent with previous studies that have demonstrated a reduction in NP size when the amount of PVA in the secondary emulsion of a double emulsion method is increased.^52^

$$Z‐\mathrm{avg}=158.61+0.03X_{1}-13.11X_{2}+4.52X_{3}-1.79X_{1}X_{2}-0.06X_{1}X_{3}-7.06X_{2}X_{3}-0.49X_{1}^{2}-3.28X_{2}^{2}+0.77X_{3}^{2}-5.16X_{1}X_{2}X_{3}-0.58X_{1}^{2}X_{2}-5.48X_{1}^{2}X_{3}+3.93X_{1}X_{2}^{2}$$

#### 3.3.2 Polydispersity Index

Having a narrow PDI in NP formulations is crucial for ensuring uniformity and consistency in particle size distribution. The PDI is a measure of the distribution of particle sizes, with lower values (<0.4) indicating a more homogeneous size distribution, while values greater than 0.4 suggesting high polydispersity.^53^ Maintaining a low PDI is essential for ensuring the ability to formulate a reproducible drug delivery system with an improved particle stability.^54^

The PDI values for the nanoparticle formulations were found to range from 0.14 to 0.32, as presented in Table 2. All independent factors were shown to have a significant effect on PDI, with *p* values of 0.0075, 0.0023, and 0.0187 for *X*_1_, *X*_2_, and *X*_3_, respectively (Figure 2). The negative coefficients of *X*_1_ and *X*_2_ in the polynomial equation suggest that the PDI value increases as the mass ratio of PEtOx-*b*-PCL/DDAB or the volume ratio of W_1_/O phase decreases, whereas the positive coefficient of *X*_3_ suggests that the PDI increases as the volume of the PVA solution increases. Both DDAB and PVA act as surfactants and stabilizing agents. Beyond certain thresholds, their increased amounts may influence interfacial tension and colloidal stability, potentially resulting in variations in particle size distribution and an elevated PDI.^55,56^ Additionally, a reduction in the ratio of W_1_/O phases in the primary emulsion may contribute to wider size distribution, as a larger organic layer requires more time to evaporate, a factor often linked to larger particles and broader size distribution.^57,58^

$$PDI=0.178-0.029X_{1}-0.038X_{2}+0.024X_{3}-0.026X_{1}X_{2}-0.0002X_{1}X_{3}-0.025X_{2}X_{3}+0.027X_{1}^{2}+0.008X_{2}^{2}+0.011X_{3}^{2}-0.006X_{1}X_{2}X_{3}+0.007X_{1}^{2}X_{2}-0.011X_{1}^{2}X_{3}+0.007X_{1}X_{2}^{2}$$

**Figure 2:**
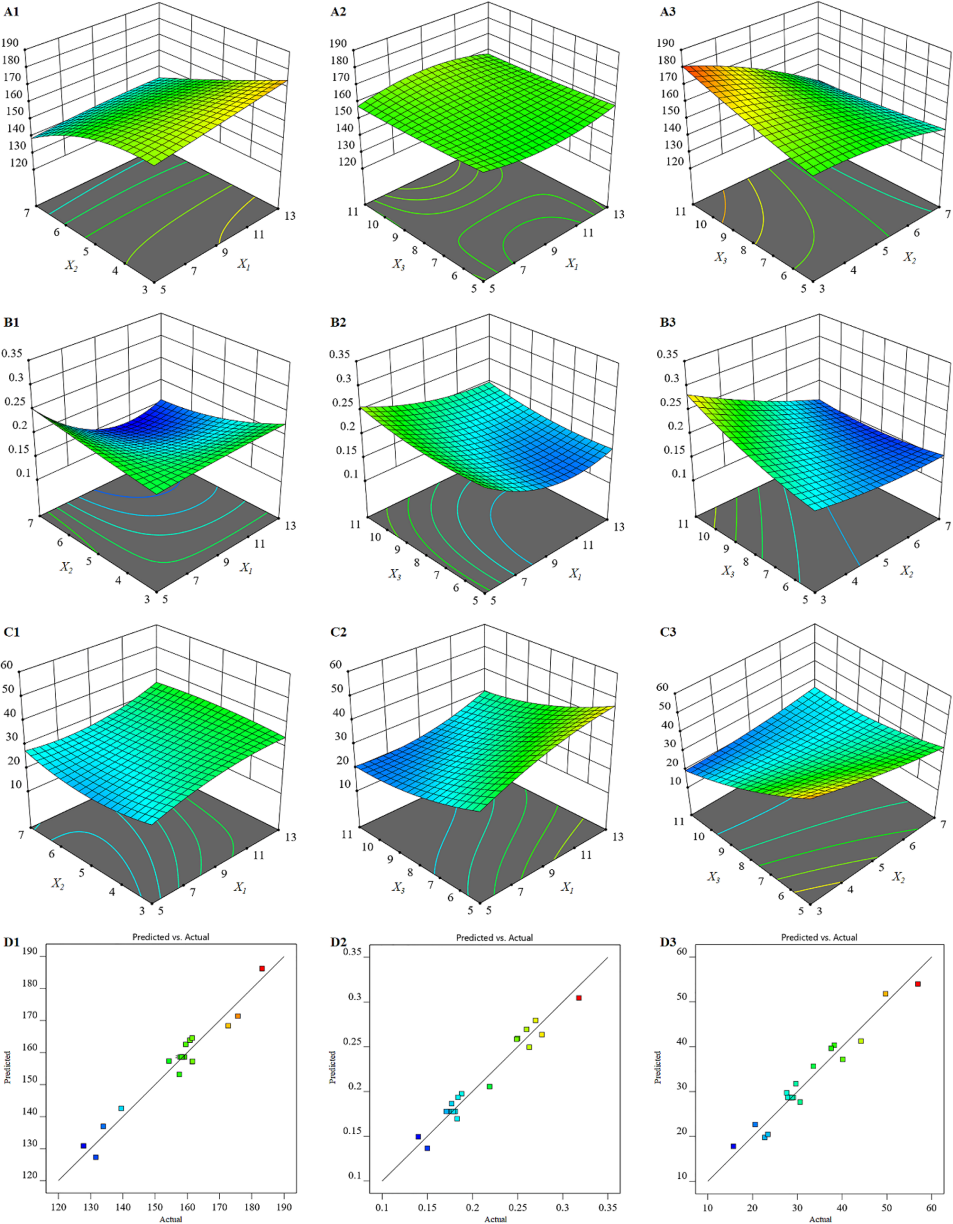
3D response surface plots displaying the effect of the mass ratio of PEtOx-*b*-PCL/DDAB, The volume ratio of *W*_2_/*O* phase, and the volume of the hardening solution on hydrodynamic diameter (A1-A3), PDI (B1–B3), and encapsulation efficiency (C1–C3). The linearity graphs of PMT-NP formulations showing the relationship between actual and predicted values for hydrodynamic diameter (D1), PDI (D2), and encapsulation efficiency (D3).

#### 3.3.3 Encapsulation Efficiency

It is important to have the highest possible % EE as it ensures that a larger amount of the drug payload is effectively incorporated into the nanoparticles, maximizing the therapeutic potential of the formulation.^59^ Nanoparticles with high encapsulation efficiency can protect the drug from degradation, control its release kinetics, and target specific sites within the body, leading to improved treatment outcomes.^60^ Additionally, high encapsulation efficiency reduces drug wastage during formulation processes, enhances drug loading capacity, and minimizes potential side effects by ensuring that a significant portion of the drug reaches the intended target.^61^

The % EE values for the PMT NP formulations ranged from 15.72% to 56.93%, as illustrated in Table 2 and Figure 2. Both independent factors, *X*_1_ and *X*_3_, exhibited a significant effect on % EE (*P*-value = 0.0074 and 0.0008, respectively), indicating an increase in % EE with higher mass ratios of PEtOx-*b*-PCL/DDAB or lower volumes of PVA solution. These results imply that reducing the surfactant amount in either the primary or secondary emulsion can enhance the PMT encapsulation efficiency in the NP formulation. Previous research has demonstrated that decreasing surfactant concentration can promote stronger interactions between hydrophobic drug molecules and the polymeric matrix of the NP system. This enhances the opportunities for the hydrophobic agent to interact with the nanoparticle core, thereby facilitating higher drug entrapment efficiency.^62^

$$\mathrm{EE}=28.69+6.39X_{1}-2.83X_{2}-9.96X_{3}-0.31X_{1}X_{2}-1.58X_{1}X_{3}+6.22X_{2}X_{3}+0.65X_{1}^{2}+1.32X_{2}^{2}+3.02X_{3}^{2}+0.07X_{1}X_{2}X_{3}+3.35X_{1}^{2}X_{2}+3.26X_{1}^{2}X_{3}-2.62X_{1}X_{2}^{2}$$

### 3.4 Design Space and Selection of the Optimal PMT NPs Parameters

The optimal parameters for PMT NPs were established through response surface analysis and design space evaluation employing a CCD method. The goal was to minimize both hydrodynamic diameter and PDI while maximizing % EE. The optimal formulation was selected by prioritizing hydrodynamic diameter and % EE, assigning them the highest importance, followed by PDI.

The selected optimal formulation exhibited an observed hydrodynamic diameter of 157.02 nm, a PDI of 0.18, and a % EE of 53.17% (Table 4). The actual values closely aligned with the predictions generated by the second-order polynomial models for all evaluated responses, reflecting a narrow 95% confidence and tolerability intervals (Table 4). The statistical analysis validated the model’s accuracy, affirming that the process of optimization, underpinned by QbD methodology, efficiently yielded PMT NPs with desirable hydrodynamic diameter, PDI, and % EE. This optimized formulation was chosen for further experimental investigations.

**Table 4:** Optimized Variables Alongside the Predicted and Actual Response Values, Including Confidence Levels and Tolerance Intervals

factor	value	response	predicted value	observed value	95% CI for mean (low–high)	95% TI for mean (low–high)
*X*_1_ (w/w)	9.87	*Y*_1_ (nm)	158.04	157.02	147.82–168.26	122.34–193.74
*X*_2_ (v/v)	3.00	*Y*_2_	0.19	0.18	0.15–0.22	0.07–0.30
*X*_3 (_mL)	5.00	*Y*_3_ (%)	52.99	53.17	45.99–60.01	28.52–77.48

### 3.5 Preparation and Characterization of GQD-Loaded PMT NPs

Different amounts of N-GQDs and S-GQDs were incorporated into the optimal formulation of PMT NPs to introduce an optically active fluorescent marker for tracking the biodistribution and cellular uptake of the nanoparticles. Varying amounts of N-GQDs or S-GQDs were added to investigate their impact on the physicochemical properties of the nanoparticles. The resulting nanoparticles, denoted as N-PMT NPs or S-PMT NPs based on the type of carbon quantum dots used, exhibited similar hydrodynamic diameter, PDI, and PMT encapsulation efficiency compared to the optimal PMT NPs (Table 5). This indicates that the introduction of carbon quantum dots did not alter the physicochemical properties of the formulation.

**Table 5:** Characterization Data after Formulating PMT NPs in the Presence of Varying Amounts of N-GQDs or S-GQDs

	hydrodynamic diameter (nm)	PDI	zeta potential (mV)	PMT encapsulation efficiency (%)	GQD encapsulation efficiency (%)
N-PMT NPs	143.5 ± 0.38	0.13 ± 0.02	6.25 ± 0.77	46.35 ± 0.08	99.15 ± 0.27
S-PMT NPs	139.6 ± 0.88	0.15 ± 0.03	8.13 ± 0.95	56.01 ± 0.16	98.83 ± 0.68

Furthermore, the different amounts of N-GQDs and S-GQDs did not lead to significant differences in the physicochemical characteristics between them (Table 5). Both N-PMT NPs and S-PMT NPs showed comparable hydrodynamic diameter and PDI, as demonstrated in Figure 3A and B. TEM images revealed spherical morphologies of comparable size for both N-PMT NPs (Figure 3D) and S-PMT NPs (Figure 3E), confirming the absence of morphological differences between the two formulations. Accordingly, both N-PMT NPs and S-PMT NPs were selected for subsequent cellular studies.

**Figure 3:**
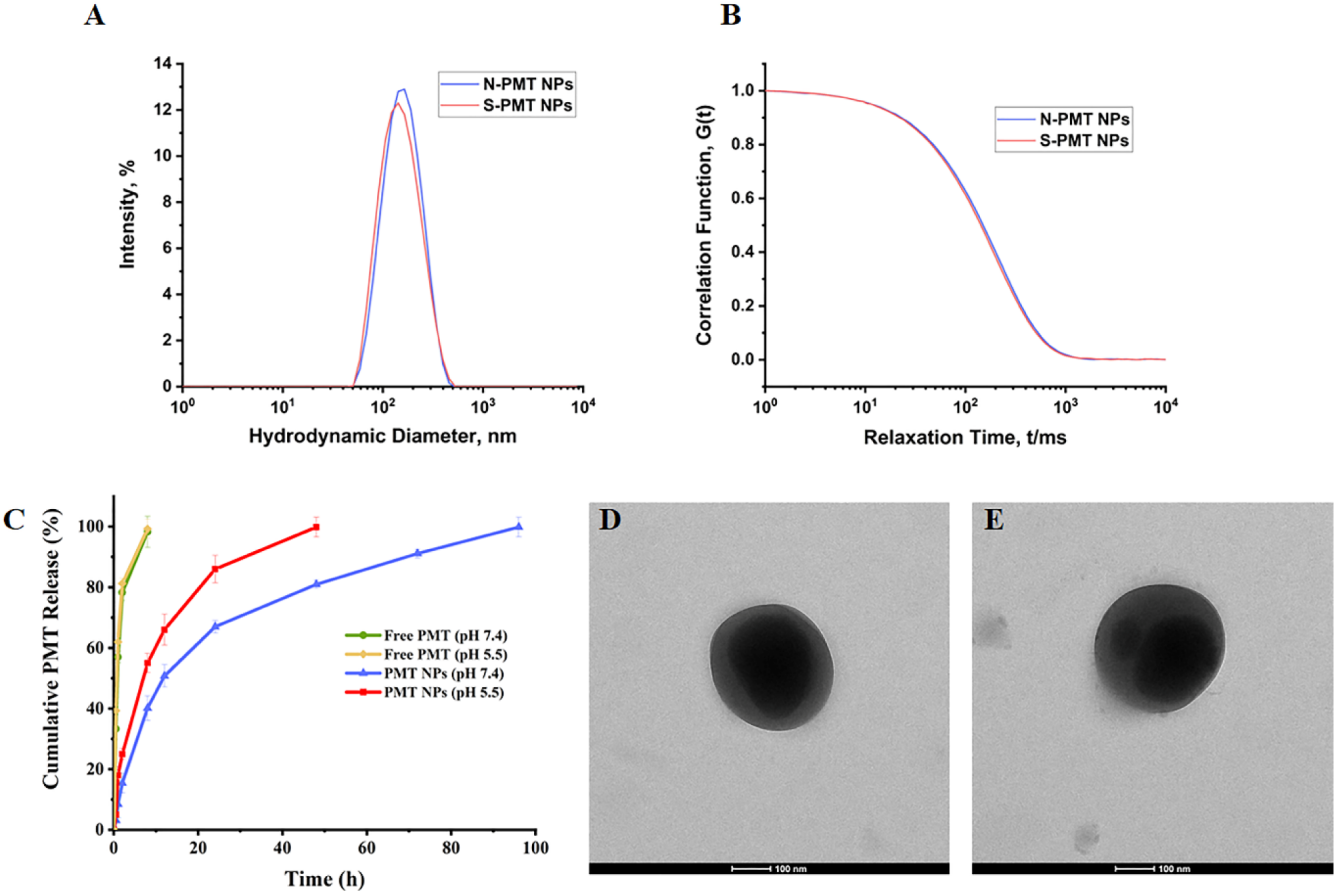
DLS results of the N-PMT NPs and S-PMT NPs, showing the hydrodynamic diameter (A) and correlation function (B) data. Release profiles of free PMT and PMT NPs at pH 5.5 and pH 7.4 (C). TEM images of N-PMT NPs (D) and S-PMT NPs (E).

The *in vitro* release data (Figure 3C) revealed a distinct difference in the release kinetics of PMT from free drug solutions and nanoparticle formulations under physiological (pH 7.4) and tumor-mimicking acidic conditions (pH 5.5). Free PMT showed a rapid burst release, with more than 50% released within the first hour at both pH levels, and nearly complete release by 8 h. In contrast, PMT NPs exhibited a modified release pattern, with only ∼8.5% and ∼18% of PMT released at 1 h under pH 7.4 and pH 5.5, respectively. Over 96 h, cumulative PMT release from NPs reached ∼67% at pH 7.4 and ∼100% at pH 5.5. The accelerated release under acidic conditions can be attributed to the ionizable nature of the PEtOx segment, which enhances polymer hydration and facilitates drug diffusion in acidic environments.^63^ This pH-responsive release profile validates the functional design of the developed nanoparticles and highlights their potential for controlled and effective drug delivery.

Additionally, to evaluate the physical stability of the NPs under physiologically relevant conditions, the optimized formulations were dispersed in DMEM and their hydrodynamic size distribution was monitored by DLS for 24 h at 37 °C. Both N-PMT NPs and S-PMT NPs showed only a slight increase in hydrodynamic diameter (from 143.5 ± 0.38 nm to 149.7 ± 1.51 nm and from 139.6 ± 0.88 nm to 144.9 ± 1.82 nm, respectively), while maintaining low PDI values (0.13 ± 0.02 to 0.20 ± 0.45 for N-PMT NPs, and 0.15 ± 0.03 to 0.19 ± 0.26 for S-PMT NPs). These results confirm that the nanocarriers retain their colloidal stability in a biologically relevant medium, supporting their robustness and reliability for subsequent *in vitro* and potential *in vivo* applications.

### 3.6 *In Vitro* Cellular Uptake and Cytotoxicity Experiments on MCF-7 Cell Lines

Conventional cancer treatments often encounter limitations such as poor aqueous solubility, inadequate targeting, broad systemic distribution, heightened toxicity, and a low therapeutic index. To overcome these challenges, this study focused on optimizing NPs based delivery of the PMT therapeutic agent. We engineered PMT NPs as a delivery platform to enhance the efficacy of PMT, specifically for intravenous administration in breast cancer therapy. The nanocarrier system was designed to improve cellular internalization of PMT and to shield the encapsulated drug from enzymatic degradation within tumor cells, thereby prolonging its retention at the tumor site. Optically active components, such as N-GQDs or S-GQDs, were integrated into the formulation to enable tracking of NPs biodistribution and cellular uptake. The MCF-7 cell line served as a 2D in vitro model to evaluate both the internalization and cytotoxic effects of the nanoparticle formulation in comparison to the free drug, with the goal of advancing PMT delivery efficiency in breast cancer treatment.

The confocal microscopy images (Figure 4A) clearly revealed the uptake of N-PMT NPs and S-PMT NPs into the MCF-7 breast cancer cells, showing their distribution in the cytoplasm. This observation indicates that the NPs can be internalized within the cells and detected due to the encapsulated N-GQDs or S-GQDs. The bioimaging capabilities of both N-PMT NPs and S-PMT NPs were similar, suggesting that the N or S doping did not alter their ability to be visualized within the cells.

**Figure 4:**
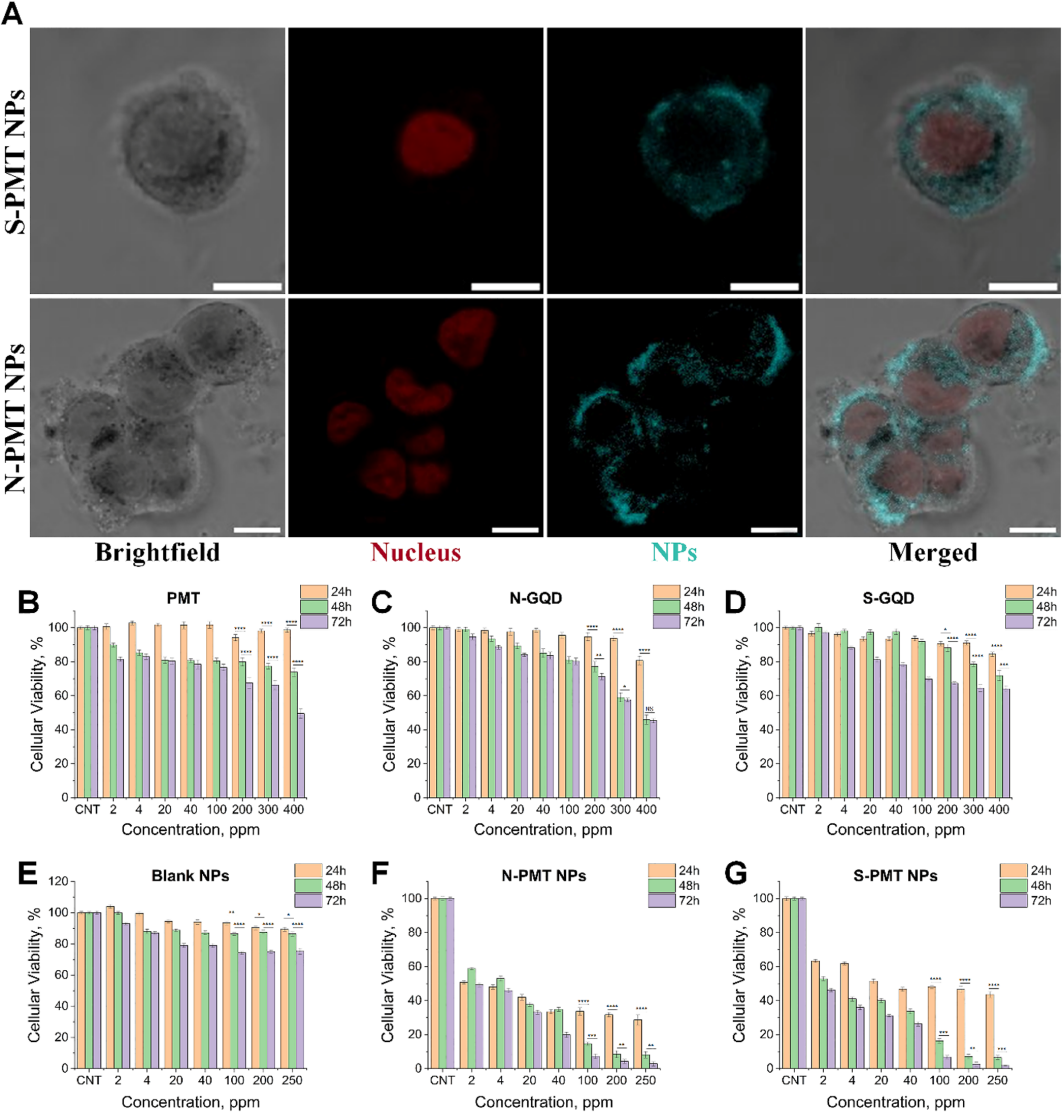
Confocal imaging of MCF-7 cells treated with S-PMT NPs, and N-PMT NPs (A) (Scale bars: 10 μm). Nuclear stain DRAQ5 is shown in red and blue for S-PMT NPs/N-PMT NPs. Cellular viability results of PMT (B), N-GQDs (C), S-GQDs (D), Blank NPs (E), N-PMT NPs (F), and S-PMT NPs (G) on MCF-7 cell line. One-way ANOVA with Tukey’s post hoc test; NS, not significant; *p* < 0.05 (*), *p* < 0.01 (**), *p* < 0.001 (***), *p* < 0.0001 (****).

Subsequent assessment of the metabolic activity of MCF-7 cells following treatment with different formulations at various time points and concentrations was conducted. Treatment with blank nanoparticles consistently resulted in a metabolic activity of over 80% (Figure 4E), indicating the low cytotoxicity and high biocompatibility of the polymers used in the nanoparticles. Free N-GQDs (Figure 4C) did not exhibit marked toxicity after 24 h across the tested concentrations, but a decrease in metabolic activity was observed after 48 h, particularly at concentrations exceeding 300 ppm, indicating a time-dependent effect. The decrease in metabolic activity was consistent between 48 and 72 h. Similarly, free S-GQDs (Figure 4D) showed no significant loss in metabolic activity at 24 h but exhibited decreased activity at 48 and 72 h, albeit to a lesser extent compared to N-GQDs, suggesting higher biocompatibility at higher concentrations.

Importantly, the cytotoxicity results of PMT NPs demonstrated a time dependent increase in antitumor activity. Both N-PMT NPs and S-PMT NPs exhibited significantly higher inhibition of MCF-7 cell proliferation at extended incubation times (48–72 h) compared to free PMT. Notably, at a concentration of 2 ppm after 72 h, N-PMT NPs and S-PMT NPs inhibited cell proliferation by 50.7% and 53.8%, respectively (Figure 4F), whereas free PMT achieved only 37.8% inhibition at the same dose. This progressive cytotoxicity correlates with the pH-dependent sustained release profile of the nanoparticles (Figure 3C). While free PMT (Figure 4B) showed a rapid burst release (>50% within 1 h at both pH 7.4 and 5.5), the nanoparticle formulations released PMT in a controlled and gradual manner, with faster release observed under acidic conditions (pH 5.5) that mimic the tumor microenvironment. These findings clearly demonstrate the improved therapeutic performance of the nanoparticle formulations compared to free PMT and are consistent with previous reports on quantum dot conjugated PMT nanomedicine,^64^ underscoring the potential of the optimized nanoparticles for enhanced drug delivery in breast cancer therapy. In addition, we recognize the need for further cellular evaluations, including quantitative analyses of uptake and cell death pathways as well as expanded cytotoxicity screening in additional breast cancer and normal cell lines, to better substantiate the selectivity and mechanistic basis of the observed therapeutic effects and enhance the translational relevance of the proposed two-in-one nanocarrier system.

## 4 Conclusions

We developed and optimized PEtOx-*b*-PCL based NPs using a statistically refined double emulsion approach. This work introduces a novel theranostic nanoparticle system that, for the first time, combines N-GQDs or S-GQDs with a PEtOx-*b*-PCL copolymer matrix for the codelivery of PMT. The optimized formulations exhibited favorable characteristics, including nanoscale hydrodynamic diameters (∼140–160 nm), low PDI, positive surface charge, and high encapsulation efficiencies (46–56% for PMT and >98% for GQDs). TEM analysis confirmed the formation of uniformly distributed, spherical nanoparticles, while in vitro studies with MCF-7 cells demonstrated efficient internalization, strong fluorescence for imaging, and significantly enhanced cytotoxic activity compared to free PMT, particularly at 2 ppm. Short-term colloidal stability was maintained for 24 h at 37 °C, confirming formulation robustness under physiological conditions. At the same time, we recognize that extended stability assessments and comprehensive in vivo immunological safety studies will be crucial to fully validate long-term safety and clinical applicability. Collectively, these results highlight the novelty and promise of this two-in-one nanocarrier system, offering an effective and integrated strategy for both cancer therapy and real-time bioimaging.

## Supplementary Material



## Notes

AFM image and height profile along the line of N-GQDs (Figure S1); XPS survey spectra of N-GQDs (Figure S2); High-resolution XPS spectra and the deconvolution of C 1s in N-GQDs (Figure S3); High-resolution XPS spectra and the deconvolution of N 1s in N-GQDs (Figure S4); High-resolution XPS spectra and the deconvolution of O 1s in N-GQDs (Figure S5); Optical properties of N-GQDs: Absorption (dash line) and emission spectra at different excitation wavelengths (color lines). (Figure S6); Normalized fluorescence spectra of N-GQDs in water at different excitation wavelengths (Figure S7); AFM image and height profile along the line of S-GQDs (Figure S8); XPS survey spectra of S-GQDs (Figure S9); High-resolution XPS spectra and the deconvolution of S 2p in S-GQDs (Figure S10); High-resolution XPS spectra and the deconvolution of C 1s in S-GQDs (Figure S11); High-resolution XPS spectra and the deconvolution of N 1s in SGQDs (Figure S12); High-resolution XPS spectra and the deconvolution of O 1s in SGQDs (Figure S13); Optical properties of S-GQDs: Absorption (dash line) and emission spectra at different excitation wavelengths (color lines) (Figure S14); Normalized fluorescence spectra of S-GQDs in water at different excitation wavelengths (Figure S15). Percentage of the atomic composition and binding energies of C, N and O in N-GQDs as determined by XPS (Table S1); Percentage of the atomic composition and binding energies of S, C, N and O in S-GQDs as determined by XPS (Table S2) (PDF)
