# Supplementary material for: Engineering Two-in-One Nanoparticles for Simultaneous Delivery of Graphene Quantum Dot and Pemetrexed
Source: ACS Omega. 2025 Oct 7;10(41):48934–47. doi: 10.1021/acsomega.5c07253 (PMC12547751; doi:10.1021/acsomega.5c07253)
Supplement: Supplementary file 1 [file ao5c07253_si_001.pdf]

ENGINEERING TWO-IN-ONE NANOPARTICLES FOR SIMULTANEOUS DELIVERY OF GRAPHENE QUANTUM DOT AND PEMETREXED

Umut Can Öz[1*], Berrin Küçüktürkmen[1], I. Jénnifer Gómez[2*], Amr Elsherbeny[3,4,5], Seda Tekneci[6,7], Özgür Eşim[8], Selin Göksever[1,7], Umut Uğur Özköse[9,10], Sevgi Gülyüz[9], Claudia Bazán-Cobelo[2], Özgür Yılmaz[9,11], Aylin Üstündağ[6], Jiřina Medalová[12], Asuman Bozkır[1], Lenka Zajíčková[13,14], Engin Er[15*]

[1] Department of Pharmaceutical Technology, Faculty of Pharmacy, Ankara University, Yenimahalle, Ankara, 06560, Türkiye

[2] CICA-Centro Interdisciplinar de Química e Bioloxía, Rúa as Carballeiras, Universidade da Coruña, A Coruña, 15071, Spain

[3] Division of Molecular Therapeutics and Formulation, School of Pharmacy, University of Nottingham, Nottingham NG7 2RD, UK

[4] bEx Vivo Cancer Pharmacology Centre, Translational Medical Sciences, Biodiscovery Institute, School of Medicine, University of Nottingham, Nottingham NG7 2UH, UK

[5] Department of Chemical Engineering and Biotechnology, University of Cambridge, Cambridge, CB3 0AS, UK

[6] Department of Pharmaceutical Toxicology, Faculty of Pharmacy, Ankara University, Yenimahalle, Ankara, 06560, Türkiye

[7] Ankara University Graduate School of Health Sciences, Diskapi, Ankara, 06110, Türkiye

[8] Department of Pharmaceutical Technology, Gulhane Faculty of Pharmacy, University of Health Sciences, Etlik, Ankara, 06010, Turkey

[9] Marmara Research Center, TUBITAK, Gebze, Kocaeli, 41470, Türkiye

[10] Department of Chemistry, Faculty of Science and Letters, Piri Reis University, Tuzla, İstanbul, 34940, Türkiye

[11] Department of Pharmaceutical Chemistry, Faculty of Pharmacy, Istanbul University, Istanbul, 34116 Türkiye

[12] Department of Experimental Biology, Faculty of Science, Masaryk University, Kamenice 5, 62500 Brno, Czech Republic

[13] Department of Condensed Matter Physics, Faculty of Science, Masaryk University, Brno, 61137, Czech Republic

[14] Central European Institute of Technology – CEITEC, Brno University of Technology, Brno, 61200, Czech Republic

[15] Department of Biotechnology, Biotechnology Institute, Ankara University, Keçiören, Ankara, 06135, Türkiye

Corresponding authors (*)

Dr. Umut Can Öz, umutcanoz@ankara.edu.tr

Dr. I. Jénnifer Gómez, i.jennifer.gomez@udc.es

Dr. Engin Er, eer@ankara.edu.tr

SUPPORTING INFORMATION

Characterization of Polymers

Proton nuclear magnetic resonance (^{1}H-NMR) and attenuated total reflectance Fourier transform infrared (ATR-FTIR) spectroscopy were employed to characterize the structure of the obtained precursors and block copolymers. ^{1}H-NMR spectra were recorded on a Varian NMR spectrometer operating at 600 MHz (599.90 MHz), with deuterated chloroform ($CDCl_3$) utilized as the solvent. Spectral interpretation and processing were figured out with MestReNova software (version 9.0.1).

Analysis of the functional group composition of the polymers was performed by ATR-FTIR spectroscopy (PerkinElmer Spectrum BX FT-IR) at the range of 4000 to 500 cm^{-1}.

Gel permeation chromatography (GPC) was utilized to determine the molecular weight characteristics, including number-average molecular weight (Mn) and dispersity (Đ), employing an Agilent 1100 system equipped with a refractive index detector. For hydrophilic PEtOx polymers, a Tosoh TSKGel G3000PWxl column (300 mm × 7.8 mm) with distilled water as the eluent was used. In contrast, the PCL homopolymer and PEtOx-*b*-PCL block copolymer samples were analyzed with a Waters Styragel column (4.6 mm i.d., 300 mm, 5 µm) and tetrahydrofuran (THF) as the mobile phase. Molecular weight calibrations were conducted employing polyethylene oxide standards (ranging from 0.106 to 1522 kDa) for PEtOx, and polystyrene standards (ranging from 0.78 to 100.0 kDa) for PCL and the block copolymer.

Characterization of N- and S-Doped Graphene Quantum Dots

The size and morphology of N-GQDs and S-GQDs were examined using atomic force microscopy (AFM). AFM imaging was conducted with a Dimension Icon instrument (Bruker, Germany) operating in tapping mode, utilizing an RTESPA-300 probe (300 kHz; 40 N/m) (Bruker, Germany). The acquired AFM images were analyzed using Gwyddion 2.52 software[1].

The chemical composition and structure of N-GQDs and S-GQDs were characterized X-ray photoelectron spectroscopy (XPS). XPS measurements were performed using an Axis Supra spectrometer (Kratos Analytical Ltd, UK). Survey XPS spectra were obtained at a

pass energy of 80 eV to determine the quantitative atomic composition. For detailed chemical group analysis, spectra of S 2p, C 1s, N 1s, and O 1s were acquired at a lower pass energy of 20 eV and analyzed using Casa XPS 2.3.17 software. The Shirley-type background was subtracted, and the spectra were fitted with a 30% Lorentzian and 70% Gaussian profile. Constraints on the full width at half maximum (FWHM) and peak positions were applied, with the binding energy calibrated using C–C/C=C groups at 284.5 eV.

The optical properties of N-GQDs and S-GQDs in water were investigated using UV-visible (UV-vis) and fluorescence spectroscopies. UV-vis absorption spectra were recorded using a Cary 8454 UV-Vis spectrometer (Agilent Technologies, USA), while fluorescence spectra were measured using an F900 Fluorescence spectrometer (Edinburgh Instruments Ltd, UK). Data processing was employed to remove Rayleigh scattering and second diffraction order induced by the grating.

SUPPORTING FIGURES AND DATA

1. N-GQDs

AFM images

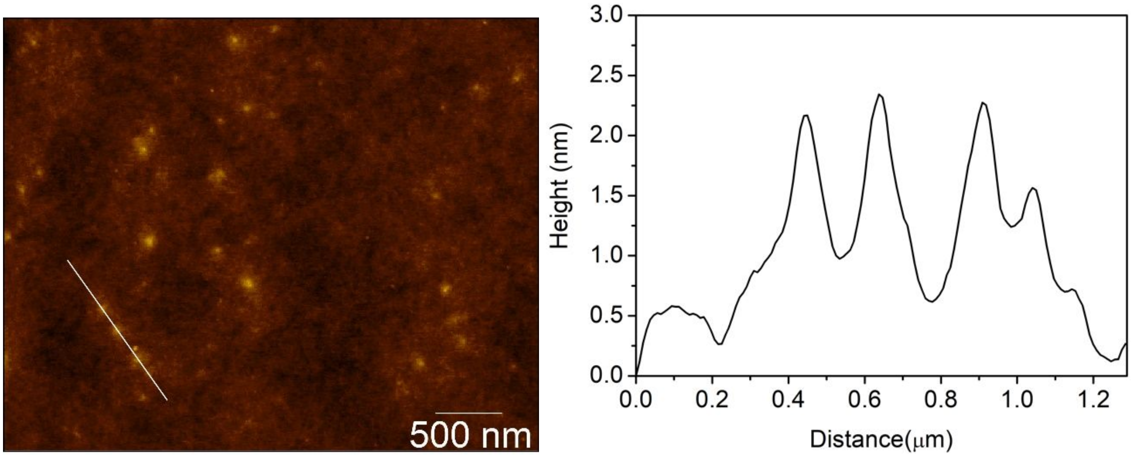

Figure S1. AFM image and height profile along the line of N-GQDs.

XPS Measurements

Table S1. Percentage of the atomic composition and binding energies of C, N and O in N-GQDs as determined by XPS.

Core level	Binding Energy (eV)	Atomic (%)
**C 1s**	**285.76**	**57.70**
C-C/C=C	284.50	33.35
C-O/C-N	285.92	53.52
C=O/C=N	287.82	12.25
COOH	288.81	0.88
**N 1s**	**399.56**	**14.40**
N=C	397.01	1.3
N-H_2/C-N-C	399.54	83.56
N-C_3	401.29	15.14
**O 1s**	**532.16**	**27.90**
O=C	530.78	12.05
O-C	532.15	79.56

C-O-C	533.47	8.39

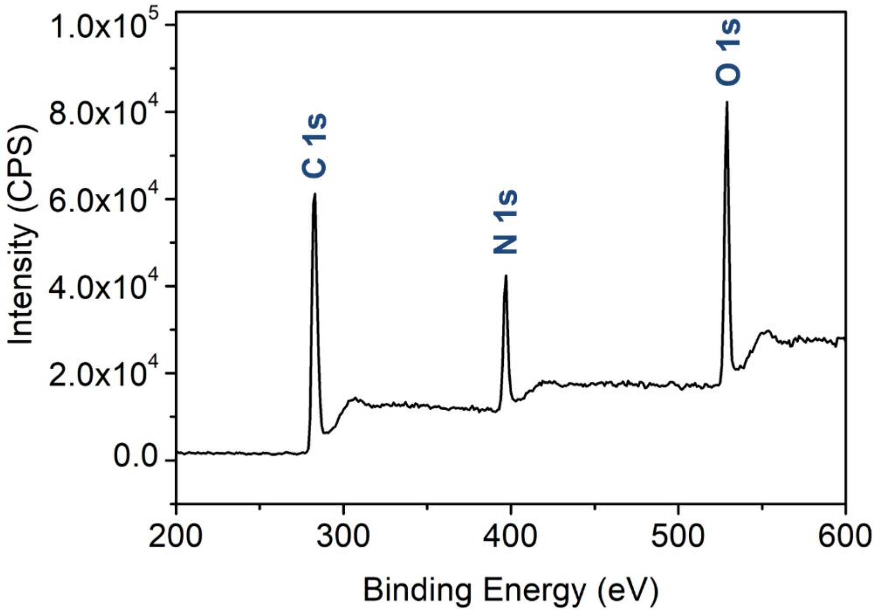

Figure S2. XPS survey spectra of N-GQDs.

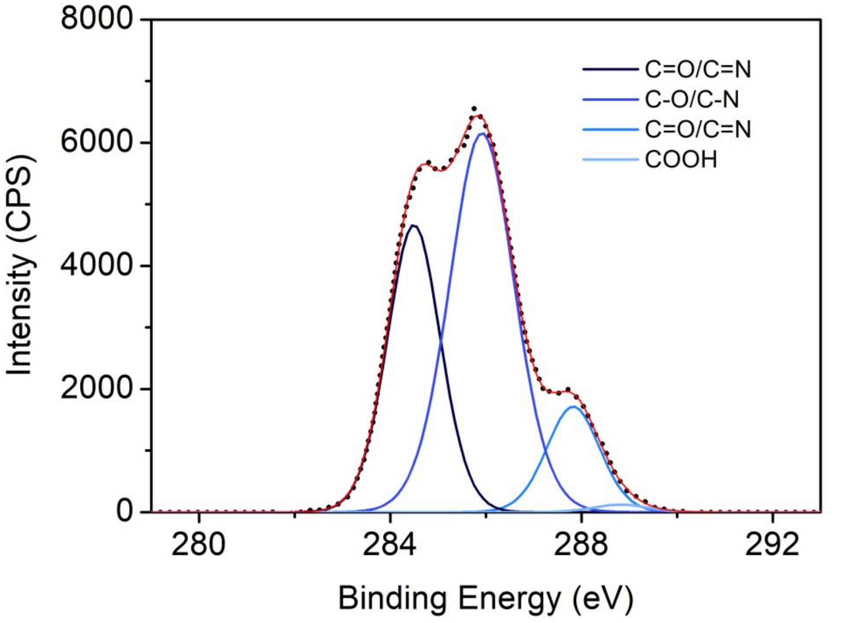

Figure S3. High-resolution XPS spectra and the deconvolution of C 1s in N-GQDs.

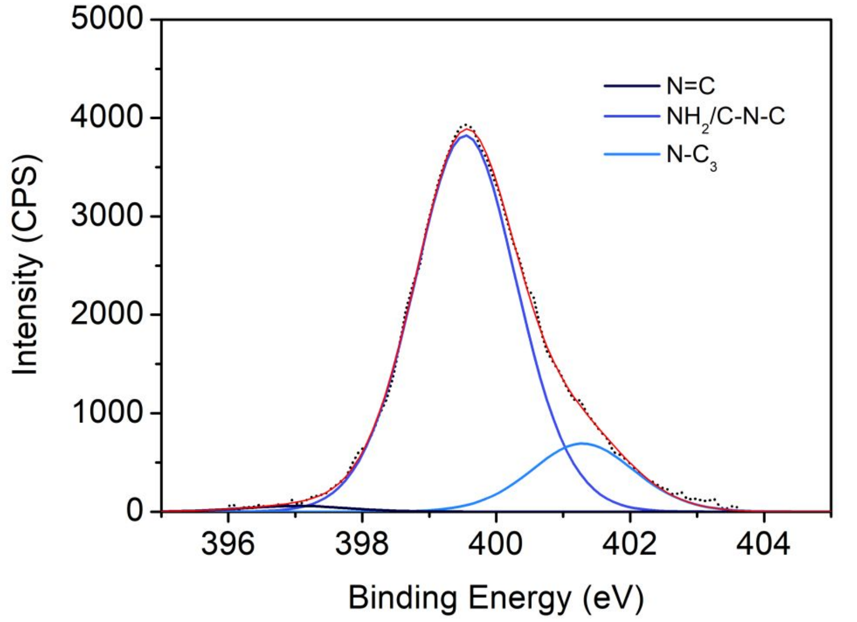

Figure S4. High-resolution XPS spectra and the deconvolution of N 1s in N-GQDs.

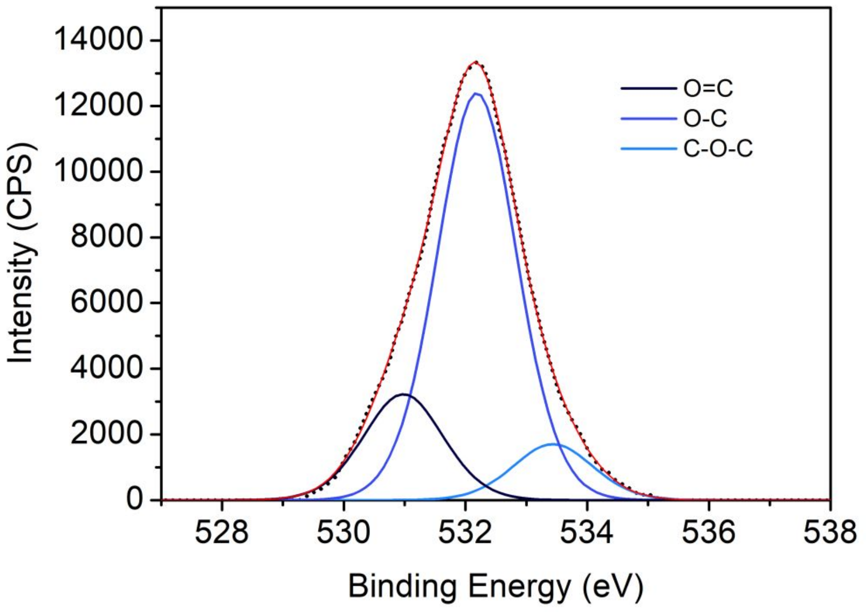

Figure S5. High-resolution XPS spectra and the deconvolution of O 1s in N-GQDs.

Optical Measurements

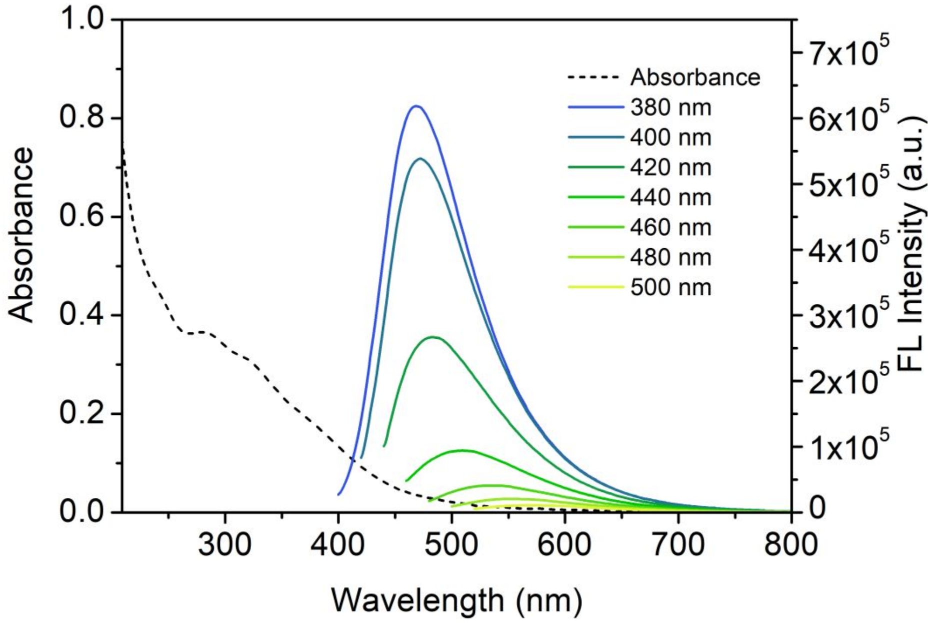

Figure S6. Optical properties of N-GQDs: Absorption (dash line) and emission spectra at different excitation wavelengths (color lines). All spectra were recorded in water at room temperature.

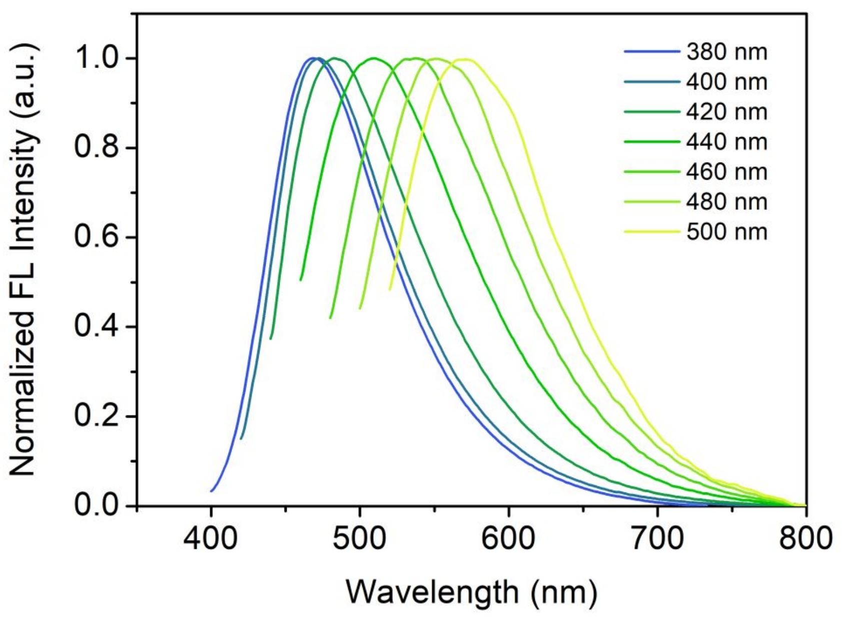

Figure S7. Normalized fluorescence spectra of N-GQDs in water at different excitation wavelengths.

2. S-GQDs

AFM images

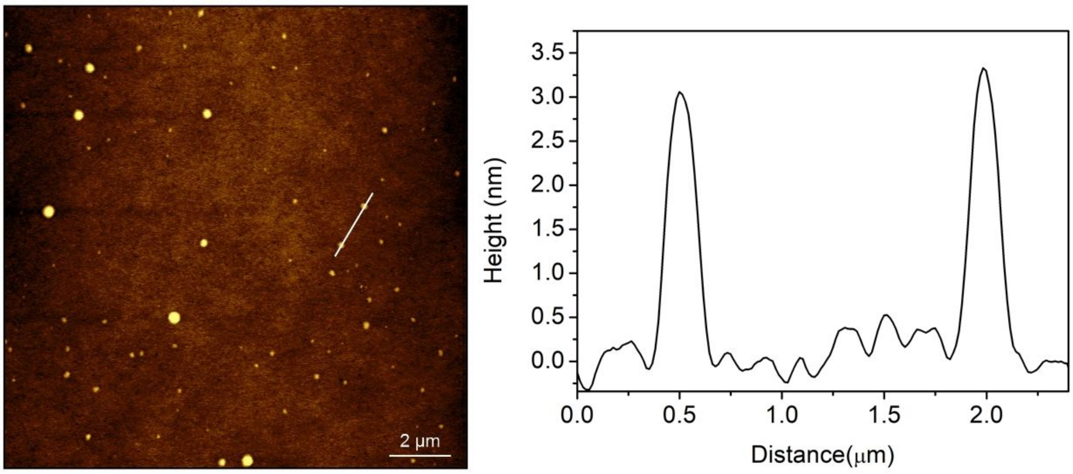

Figure S8. AFM image and height profile along the line of S-GQDs.

XPS measurements

Table S2. Percentage of the atomic composition and binding energies of S, C, N and O in S-GQDs as determined by XPS.

**Core level**	**Binding Energy (eV)**	**Atomic (%)**
**S 2p**	**163.13**	**4.1**
**C 1s**	**285.73**	**68.9**
C-C/C=C	284.50	41.1
C-O/C-N/C-S	285.95	50.5
C=O/C=N	287.51	8.4
**N 1s**	**401.43**	**5.7**
N-H_2/C-N-C	399.10	57.7
N-C_3	401.51	42.3
**O 1s**	**532.23**	**21.3**
O=C	532.27	74.1
O-C	533.61	25.9

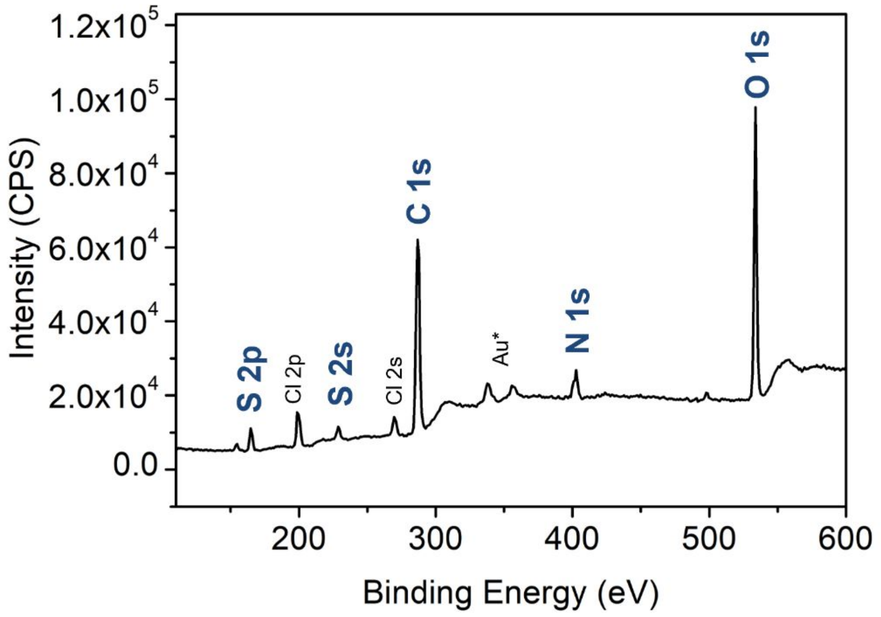

Figure S9. XPS survey spectra of S-GQDs. (Cl comes from the hydrochloride salt and Au* from the substrate)

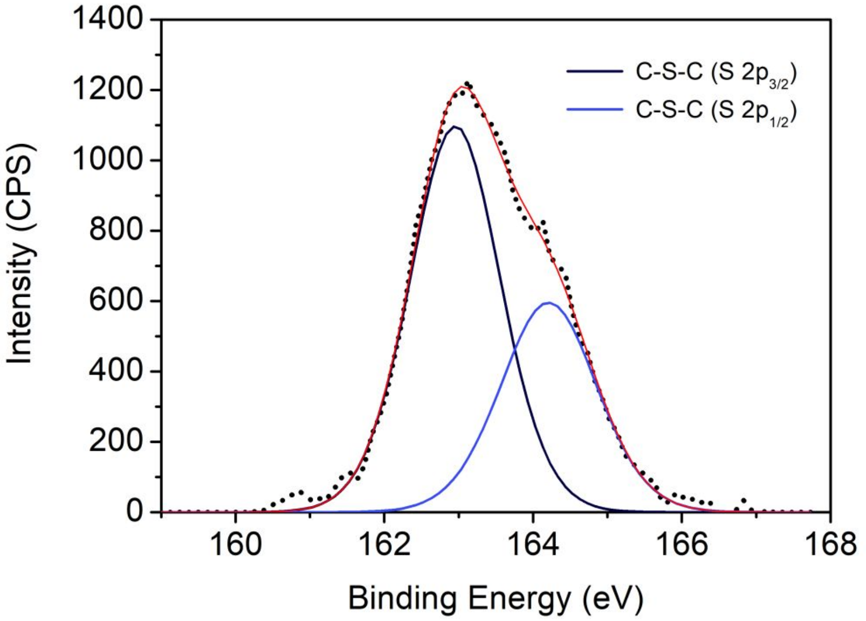

Figure S10. High-resolution XPS spectra and the deconvolution of S 2p in S-GQDs.

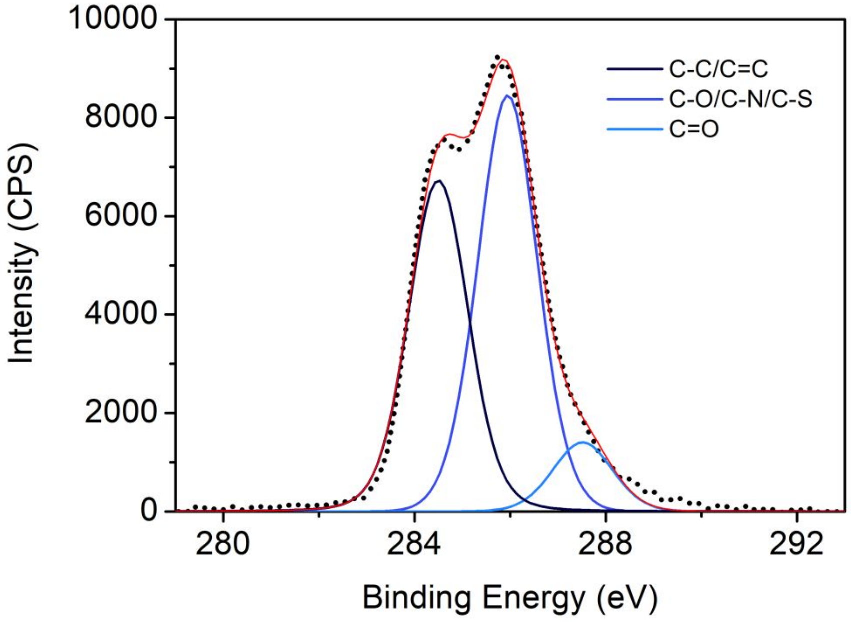

Figure S11. High-resolution XPS spectra and the deconvolution of C 1s in S-GQDs.

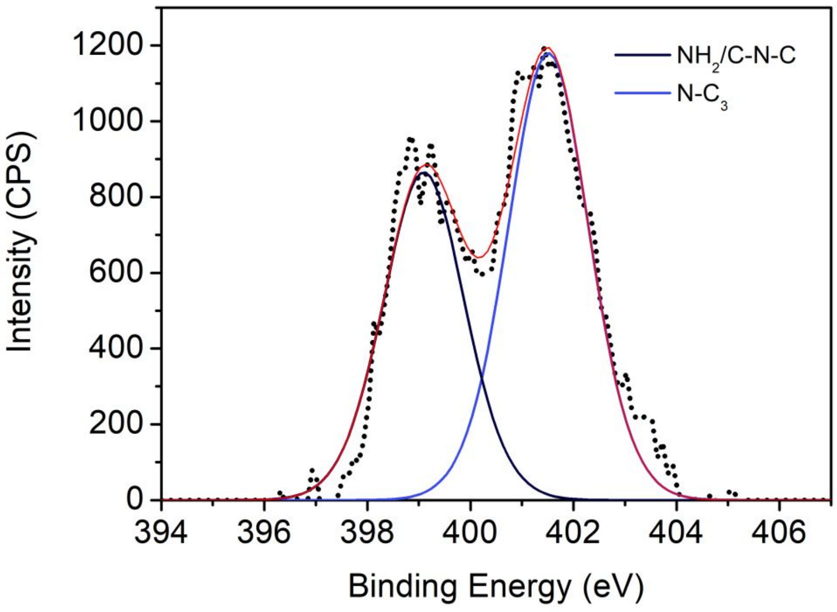

Figure S12. High-resolution XPS spectra and the deconvolution of N 1s in SGQDs.

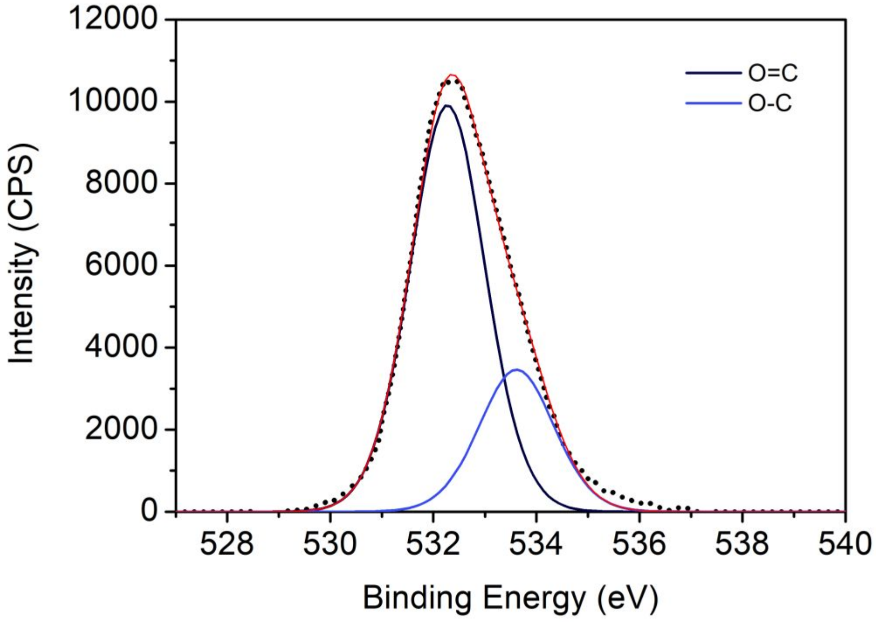

Figure S13. High-resolution XPS spectra and the deconvolution of O 1s in SGQDs.

Optical Measurements

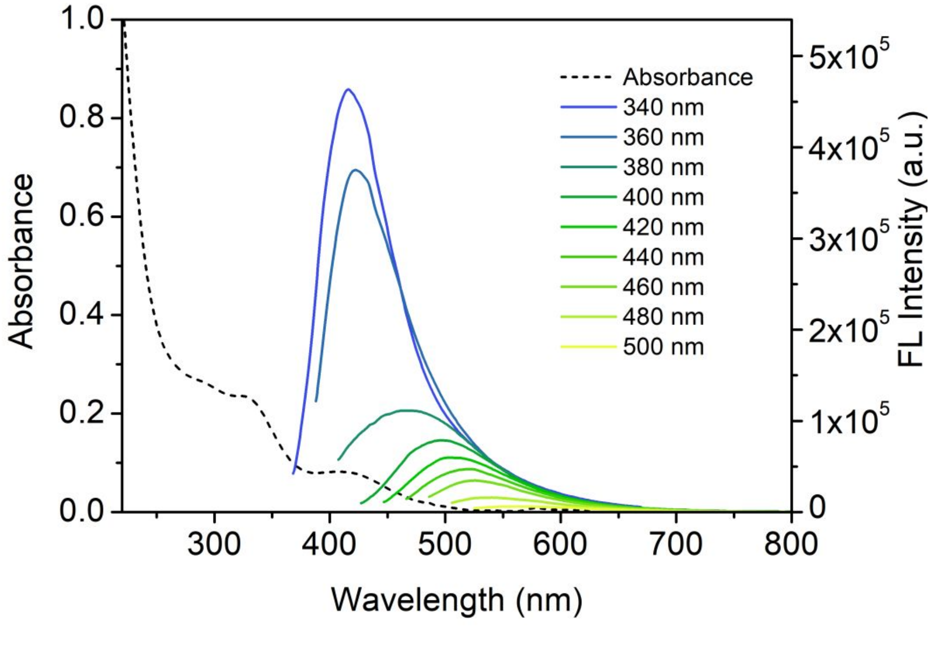

Figure S14. Optical properties of S-GQDs: Absorption (dash line) and emission spectra at different excitation wavelengths (color lines). All spectra were recorded in water at room temperature.

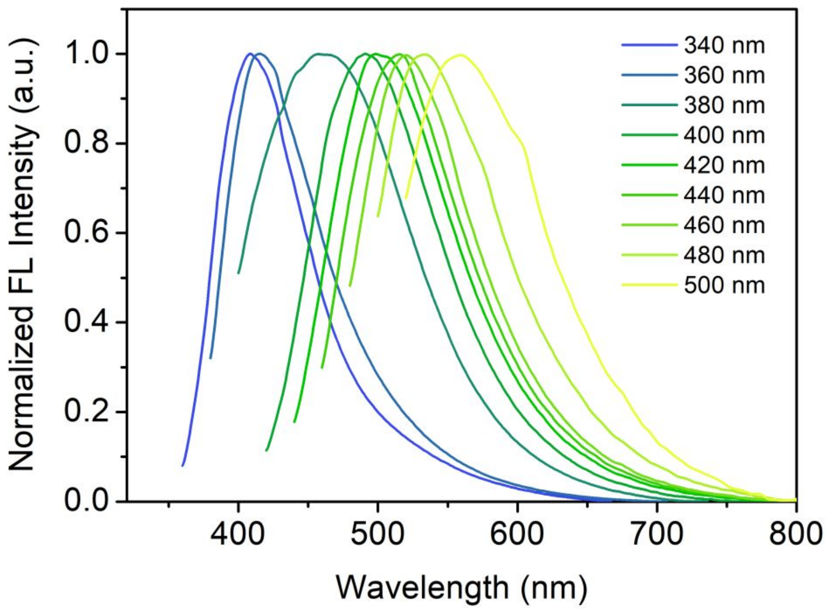

Figure S15. Normalized fluorescence spectra of S-GQDs in water at different excitation wavelengths.

References

(1) Nečas, D.; Klapetek, P. Gwyddion: an open-source software for SPM data analysis. *Open Physics* **2012**, *10* (1), 181-188. DOI: 10.2478/s11534-011-0096-2.
